# Effect of exercise training interventions on energy intake and appetite control in adults with overweight or obesity: A systematic review and meta‐analysis

**DOI:** 10.1111/obr.13251

**Published:** 2021-05-05

**Authors:** Kristine Beaulieu, John E. Blundell, Marleen A. van Baak, Francesca Battista, Luca Busetto, Eliana V. Carraça, Dror Dicker, Jorge Encantado, Andrea Ermolao, Nathalie Farpour‐Lambert, Adriyan Pramono, Euan Woodward, Alice Bellicha, Jean‐Michel Oppert

**Affiliations:** ^1^ Appetite Control and Energy Balance Research Group (ACEB), School of Psychology, Faculty of Medicine and Health University of Leeds Leeds UK; ^2^ NUTRIM School for Nutrition and Translational Research in Metabolism, Department of Human Biology Maastricht University Maastricht The Netherlands; ^3^ Sport and Exercise Medicine Division, Department of Medicine University of Padova Padova Italy; ^4^ Obesity Management Task Force (OMTF) European Association for the Study of Obesity (EASO) Teddington UK; ^5^ Department of Medicine University of Padova Padova Italy; ^6^ Faculdade de Educação Física e Desporto CIDEFES, Universidade Lusófona de Humanidades e Tecnologias Lisbon Portugal; ^7^ Department of Internal Medicine D, Hasharon Hospital, Rabin Medical Center, Sackler School of Medicine Tel Aviv University Tel Aviv Israel; ^8^ APPsyCI – Applied Psychology Research Center Capabilities & Inclusion ISPA – University Institute Lisbon Portugal; ^9^ Obesity Prevention and Care Program Contrepoids, Service of Therapeutic Education for Chronic Diseases, Department of Community Medicine, Primary Care and Emergency University Hospitals of Geneva and University of Geneva Geneva Switzerland; ^10^ INSERM, Nutrition and obesities: systemic approaches, NutriOmics Sorbonne University Paris France; ^11^ UFR SESS‐STAPS University Paris‐Est Créteil Créteil France; ^12^ Assistance Publique‐Hôpitaux de Paris (AP‐HP), Pitié‐Salpêtrière hospital, Department of Nutrition, Institute of Cardiometabolism and Nutrition Sorbonne Université Paris France

**Keywords:** appetite control, energy intake, exercise, physical activity

## Abstract

This systematic review examined the impact of exercise training interventions on energy intake (EI) and appetite control in adults with overweight/obesity (≥18 years including older adults). Articles were searched up to October 2019. Changes in EI, fasting appetite sensations, and eating behavior traits were examined with random effects meta‐analysis, and other outcomes were synthesized qualitatively. Forty‐eight articles were included (median [range] BMI = 30.6 [27.0–38.4] kg/m^2^). Study quality was rated as poor, fair, and good in 39, seven, and two studies, respectively. Daily EI was assessed objectively (*N* = 4), by self‐report (*N* = 22), with a combination of the two (*N* = 4) or calculated from doubly labeled water (*N* = 1). In studies rated fair/good, no significant changes in pre‐post daily EI were found and a small but negligible (SMD < 0.20) postintervention difference when compared with no‐exercise control groups was observed (five study arms; MD = 102 [1, 203] kcal). There were negligible‐to‐small pre‐post increases in fasting hunger and dietary restraint, decrease in disinhibition, and some positive changes in satiety and food reward/preferences. Within the limitations imposed by the quality of the included studies, exercise training (median duration of 12 weeks) leads to a small increase in fasting hunger and a small change in average EI only in studies rated fair/good. Exercise training may also reduce the susceptibility to overconsumption (PROSPERO: CRD42019157823).

## 1 INTRODUCTION

It is widely accepted that physical activity is an important component of health and obesity management.^1^, ^2^ Evidence demonstrates that the degree of success of weight loss and weight loss maintenance in people living with obesity is related to the amount of physical activity performed measured by minutes spent or energy expended.^3^, ^4^ Short‐term controlled trials with supervised regular exercise can show clear (but modest) effects on average loss of body weight and adipose tissue.^5^ However, weight loss (or fat loss) cannot be guaranteed, and the average fat loss in these trials usually masks a wide individual range of values with some participants losing, for example, three times the average, others maintaining weight, and a certain proportion even gaining weight.^6^, ^7^, ^8^ Even in some studies that show a positive effect of exercise intervention on weight loss, the degree of weight loss actually observed is often less than the weight loss theoretically expected based on the amount of energy expended.^9^ All of these outcomes depend on a number of factors, including the complex effects of exercise on physiology.^10^

However, the most salient factor determining weight change is that exercise, while obviously raising energy expenditure, also exerts an action on energy intake. The idea that energy expenditure influences appetite control was postulated more than 50 years ago by Edholm et al.,^11^, ^12^ who argued that “the differences between the intakes of food must originate in the differences in energy expenditure.”^11,p. 297^ In the last 10 years, it has been well documented that energy expenditure is a major driver of energy intake.^13^, ^14^ Although the major energy demand is generated by resting metabolic rate (RMR),^15^, ^16^ activity energy expenditure also exerts a positive but weaker effect,^17^ and daily physical activity is associated with daily food intake.^18^ These observations draw attention to the fact that exercise has an effect on both sides of the energy balance equation: energy expenditure and energy intake. Indeed, in their review, Thomas et al.^9^ conclude that “the small magnitude of weight loss observed from the majority of evaluated exercise interventions is primarily due to low doses of prescribed exercise energy expenditures compounded by a concomitant increase in caloric intake.” The ultimate effect of exercise on body fat will depend therefore on the balance between these two forces, and their capacity to generate a negative energy balance. In turn, this outcome will be influenced by a complex interaction between many factors (physiological and environmental). This account provides a background for considering the potential effects of exercise on appetite control and ultimately on body fat. However, it is recognized that a relationship between a habitual daily level of activity (as part of a permanent active lifestyle in lean active people) and daily energy intake cannot be directly compared with the effects of an imposed exercise regime in inactive individuals with obesity.^19^ This review is not concerned with the general relationship between energy expenditure and energy intake (see Blundell et al.^20^ for review) but is restricted to an examination of the effects of deliberate and imposed (i.e., prescribed) exercise regimes of fixed durations on energy intake in people with overweight or obesity. Research suggests that effects observed may be increases, decreases, or no effect^21^—depending on a complex set of prevailing circumstances. In their systematic review, Donnelly et al.^21^ found that among 36 exercise intervention studies (not limited to people with overweight or obesity) ranging between 3 and 44 weeks for nonrandomized and 12 and 72 weeks for randomized trials, 92% of nonrandomized and 75% of randomized trials reported no effect of exercise training on energy intake.

In the context of the European Association for the Study of Obesity Physical Activity Working Group, the primary aim of this systematic review was to examine the impact of exercise training interventions on energy intake and appetite control (appetite sensations, eating behavior traits, and food reward) in individuals with overweight or obesity. A secondary aim was to examine the effects of different training modalities (aerobic training, high‐intensity interval training [HIIT], resistance training, combination of aerobic and resistance training) on energy intake and appetite control.

## 2 METHODS

This systematic review follows the Preferred Reporting Items for Systematic Reviews and Meta‐Analysis (PRISMA) guidelines and is registered in the PROSPERO database (registration number CRD42019157823).

### 2.1 Search strategy

Four electronic databases (PubMed, Web of Science, Cochrane Library, and EMBASE) were searched for original articles published up to October 11, 2019 using the strategy “obesity AND physical activity AND age AND energy intake AND appetite control.” Previous systematic reviews were screened to identify relevant subject headings and key words to include within each subject category. The specific key words used for the search are listed in Table S1. Limits were set to include articles published in English. Reference lists from the resulting reviews and articles were also screened to identify additional articles.

### 2.2 Study selection, inclusion, and exclusion

Articles were included if they involved adults (≥18 years including older adults) with overweight (BMI ≥ 25 kg/m^2^) or obesity (BMI ≥ 30 kg/m^2^) participating in physical activity interventions, that is, exercise training. Studies focusing on the primary prevention of weight gain/obesity were not included. The presence of the following obesity comorbidities was not an exclusion criterion: type 2 diabetes, hypertension, dyslipidemia, metabolic syndrome, liver disease (NAFLD/NASH), and osteoarthritis. Those with the following comorbidities were excluded: cardiovascular disease (coronary artery disease, stroke, heart failure), cancers, rheumatoid arthritis, inflammatory bowel disease, kidney failure, neuropathy, severe orthopedic disorders (with important mobility limitations), intellectual deficiency, psychiatric conditions, fibromyalgia, asthma, and sleep disorders. No minimum intervention length criterion was applied. Exercise training programs included sessions with one or more types of exercise (aerobic and/or resistance and/or HIIT). Exercise sessions could be supervised, partially supervised, or nonsupervised. Only exercise training interventions were included as the combination with other interventions (e.g., diet and cognitive behavioral therapy) may influence energy intake and/or appetite control. Additionally, only exercise training interventions where diet was free to vary were included in the energy intake analysis. Comparators included no‐exercise controls. Abstracts and full texts were assessed for eligibility independently by two authors (KB and JB) with uncertainty regarding eligibility discussed among authors.

### 2.3 Data extraction and synthesis

Data were extracted by two authors (KB and JB) using standardized forms. The characteristics of each included article included reference, study design, number of participants included in intervention and control groups, population characteristics (age, BMI, % female, comorbidities for intervention and control groups), description of intervention (program duration, number of sessions/week, type of training, supervision/delivery), comparison, setting (laboratory or free‐living), outcomes, and duration of follow‐up.

The findings pertaining to energy intake, appetite sensations, eating behavior traits, or food reward of each included article are reported. In addition, the study author's conclusion was extracted, and the current authors' assessment of conclusion is provided for each included article.

Effects on energy intake were examined using random effects meta‐analysis (Comprehensive Meta‐Analysis version 3, New Jersey, USA). A combined analysis on test meal energy intake and daily energy intake was performed with standardized mean difference (SMD), whereas another analysis specifically for daily energy intake was performed with mean difference (MD). Effect sizes are reported alongside their 95% confidence intervals and *p* values. Effect sizes were considered large, medium, small, and negligible when SMD was >0.8, between 0.5 and 0.8, between 0.2 and 0.5, and <0.2, respectively.^22^ Heterogeneity was assessed using the *I*‐squared statistic (*I* ^2^), with values interpreted as low (<25%), moderate (25%–75%), and high (>75%).^23^ Publication bias was assessed with visual inspection of the funnel plot, Egger's regression test, and Duval and Tweedie's trim‐and‐fill method. Sensitivity analysis with the one‐study‐removed procedure was also performed. Medians (IQR) were converted into means (SD) using the Excel spreadsheet from Wan et al.^24^ One study^25^ reported geometric mean as opposed to arithmetic mean. Energy intakes reported in joules were converted into kilocalories. Confidence intervals were converted into SD using the Cochrane handbook formula.^26^ Data from figures were extracted in duplicate using an online tool (WebPlotDigitizer; https://automeris.io/WebPlotDigitizer/). When SD of change was not provided in addition to SD baseline and postintervention^26^ or raw data not available, a pre‐post correlation coefficient of 0.6 was used as a conservative estimate based on the calculated coefficients (range 0.49–0.86). The authors were contacted if required data were not reported in the articles. In the studies reporting data for both test meal and daily energy intake^7^, ^27^, ^28^ or from subgroups in addition to the original groups,^29^, ^30^ or from cross‐over studies,^31^, ^32^ sample size was halved to avoid “double counting” of participants in the overall analyses (full sample sizes were used for the daily energy intake analysis when test meal data were omitted). Some studies in the systematic review were not included in the meta‐analysis due to inclusion of the same data in a later study and/or in a larger sample size. Two approaches were used: one for pre‐post changes in energy intake in the exercise groups only and another for the comparison of postintervention energy intake in the exercise compared with control groups. There were not enough studies to perform subgroup analyses on exercise mode, but exploratory subgroup moderation analyses were performed, when ≥5 effect sizes were available per subgroup, to examine the effects of sex, exercise dose/intensity, and energy intake methods. A restricted maximum likelihood random‐effects meta‐regression was performed to assess whether intervention duration influenced effects on energy intake. Effects on fasting hunger and fullness, restraint, disinhibition/uncontrolled eating, and susceptibility to hunger scores are reported via meta‐analysis on pre‐post changes in exercise groups only as not enough data were available for comparisons with no‐exercise control groups. Other appetite‐related outcomes, such as food reward, are reported as a qualitative synthesis, as assessment methods and study designs varied quite markedly between studies.

### 2.4 Quality assessment

To assess study quality, we used the tool developed by the National Heart, Lung, and Blood Institute (USA) that has been previously used for defining guidelines for the management of obesity.^33^ The original assessment forms for controlled trials and single‐group intervention studies were used, and an adapted form was used for cross‐over trials based on the one for controlled trials. Four assessment items represented fatal flaws if answered “No/Not reported/Can't determine”: for controlled/cross‐over trials (i) randomization (#1), (ii) dropout rate <20% (#7), (iii) valid/reliable outcome measures (#11), (iv) intent‐to‐treat analysis (#14); and for single‐group interventions (i) eligibility criteria pre‐specified (#2), (ii) sufficient sample size (#5), (iii) valid/reliable outcome measures (#7), (iv) drop‐out rate <20% or intent‐to‐treat analysis (#9). A global rating was determined based on the number of fatal flaws: good quality (0 fatal flaws), fair quality (1 fatal flaw), or poor quality (≥2 fatal flaws). Quality assessment was conducted independently by two reviewers (KB and JB). Any disagreement between the reviewers was resolved through discussion (with a third author where necessary).

## 3 RESULTS

**FIGURE 1:**
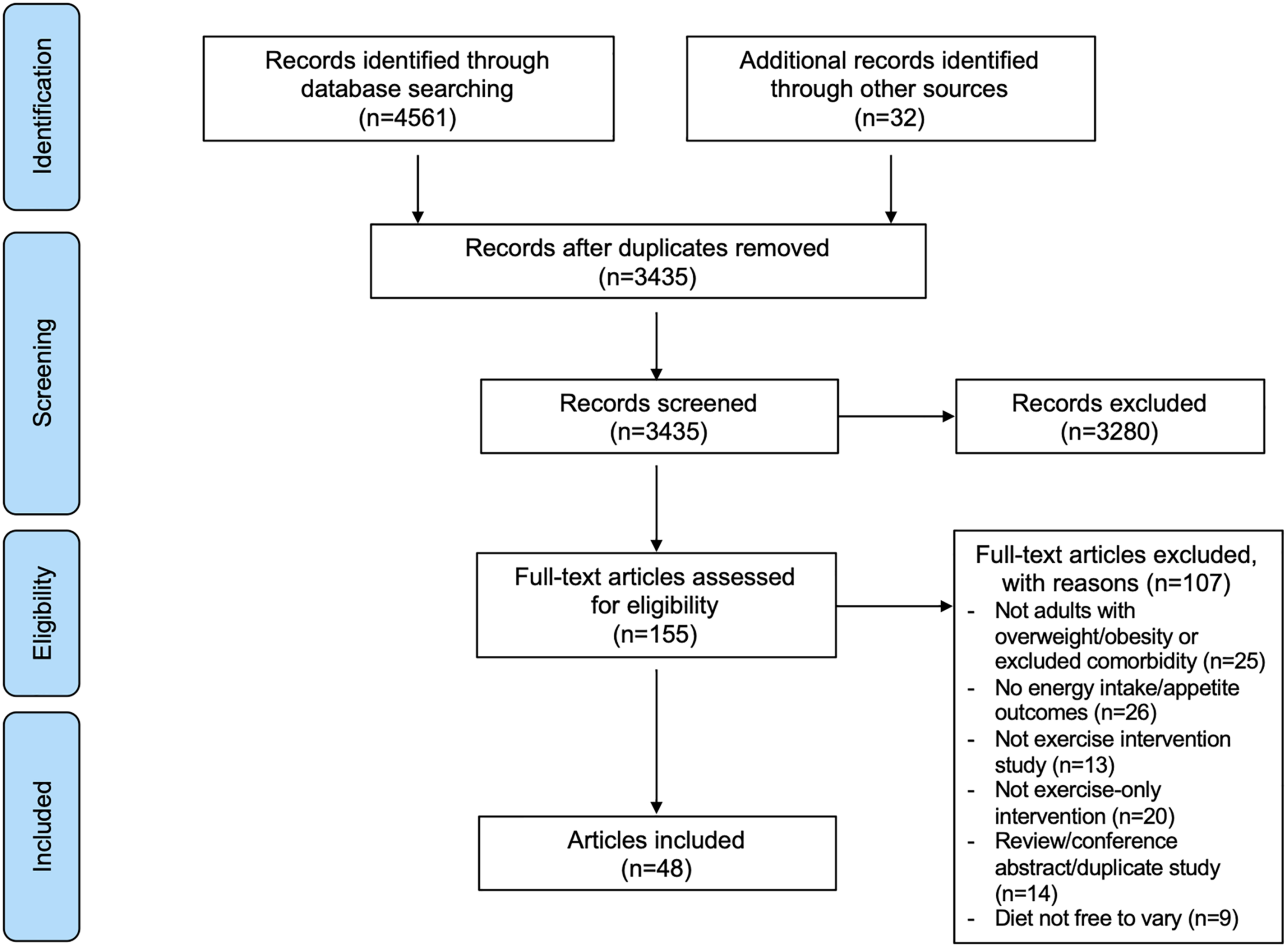
PRISMA flow‐chart

Figure 1 illustrates the systematic review flow diagram. The database search yielded 4561 articles, 3280 of which were eliminated based on titles and abstracts alone. The full text was retrieved from 155 articles and 48 satisfied the inclusion criteria.

### 3.1 Study characteristics

The characteristics of the included studies are presented in Table 1.

**TABLE 1:** Characteristics of included studies

Reference	Study design	Population	Intervention	Comparison	Setting and outcomes	Follow‐up duration
**Randomized and nonrandomized controlled trials**						
Alizadeh et al. (2017)	Randomized trial	Intervention group 1 (morning exercise): *N* = 23 Age: 34 (6) years BMI: 27.3 (1.5) kg/m^2^ % female: 100 Comorbidities: none Intervention group 2 (evening exercise): *N* = 19 Age: 34 (7) years BMI: 27.6 (1.4) kg/m^2^ % female: 100 Comorbidities: none	‐ **Program duration** **:** 6 weeks **‐Number of sessions/week:** 3 days/week **‐ Type of training:** morning (8–10 a.m.) vs. evening (2–4 p.m.) aerobic exercise (30 min treadmill running at ventilatory threshold heart rate). Education about healthy nutrition provided at baseline. **‐ Supervision:** unclear; participants performed exercise at the department of sports medicine.	Morning vs. evening exercise	**Setting:** free‐living **Outcomes:** **‐** Prospective food consumption, fullness, hunger, satiety, desire to eat savory, sweet, salty and fatty foods (VAS; completed before exercise session and 15 min after, at baseline, week 3 and week 6) **‐** Self‐reported food intake (24‐h food record before and after exercise sessions at baseline, week 3 and week 6)	No follow‐up; just baseline, week 3 and postintervention measurements
Bales et al. (2012)	Randomized trial	Intervention group 1 (aerobic training; AT): *N* = 39 Age: 53 (45, 57) years^a^ BMI: 30.1 (27.8, 32.6) kg/m^2^ ^a^ % female: 51% Comorbidities: dyslipidemia Intervention group 2 (resistance training; RT): *N* = 38 Age: 49 (42, 59) years^a^ BMI: 30.4 (28.6, 33.4) kg/m^2^ ^a^ % female: 53% Comorbidities: dyslipidemia Intervention group 3 (aerobic + resistance training; AT + RT): *N* = 40 Age: 47 (41, 55) years^a^ BMI: 30.2 (27.6, 33.4) kg/m^2^ ^a^ % female: 55% Comorbidities: dyslipidemia	**‐Program duration:** 8 months **‐ Number of sessions/week**: AT: calorically equivalent to ~20 km/week (frequency NR) RT: 3 days/week AT + RT: linear combination **‐ Type of training:** AT: prescribed number of minutes each week (NR), treadmill, elliptical, cycle ergometers at 65%–85% VO_2peak_ RT: 3 sets, 8–12 reps/set, all major muscle groups targeted, weight increased by 2.2 kg when appropriate **‐ Supervision:** yes; AT: by staff, RT: staff and/or “FitLinxx Strength Training Partner”	Aerobic training vs. resistance training vs. aerobic + resistance training	**Setting:** free‐living **Outcomes:** **‐** Self‐reported food intake (3‐d food records and 24‐h diet recall; “quantitative daily dietary intake”)	No follow‐up; just baseline and postintervention measurements
Beaulieu et al. (2020)	Non‐RCT	Intervention group: *N* = 46 Age: 43 (8) years BMI: 30.5 (3.8) kg/m^2^ % female: 65% Comorbidities: none Control group: *N* = 15 Age: 41 (11) years BMI: 31.4 (3.7) kg/m^2^ % female: 60% Comorbidities: none	**‐Program duration:** 12 weeks **‐Number of sessions/week:** 5 days/week **‐ Type of training:** aerobic exercise (500 kcal at 70% HR_max_) **‐ Supervision:** yes; research staff	Exercise vs. no‐exercise control (recruited separately) *Control group instructed to continue usual dietary and exercise habits*	**Setting**: laboratory (high‐fat and high‐carbohydrate food probe days) **Outcomes:** **‐** Liking and wanting for high‐fat relative to low‐fat foods (Leeds Food Preference Questionnaire; LFPQ) **‐** Restraint, disinhibition, susceptibility to hunger (TFEQ), binge eating	No follow‐up; just baseline and postintervention measurements
Bhutani et al. (2013)	RCT	Intervention group 1 (alternate day fasting + endurance exercise; ADF + EX): *N* = 18 (16 completers) Age: 45 (21) years BMI: 35 (4) kg/m^2^ % female: 100% Comorbidities: none Intervention group 2 (alternate day fasting; ADF): *N* = 25 (16 completers) Age: 42 (10) years BMI: 35 (5) kg/m^2^ % female: 96% Comorbidities: none Intervention group 3 (endurance exercise; EX): *N* = 24 (16 completers) Age: 42 (10) years BMI: 35 (5) kg/m^2^ % female: 96% Comorbidities: none Control group: *N* = 16 Age: 49 (8) years BMI: 35 (4) kg/m^2^ % female: 94% Comorbidities: none	**‐Program duration:** 12 weeks **‐Number of sessions/week:** 3 days/week **‐ Type of training:** aerobic exercise, starting at 25 min at 60% HR_max_, building up to 40 min at 75% HR_max_. **‐ Supervision:** yes; exercise performed at the research center	Alternate day fasting + endurance exercise vs. alternate day fasting vs. endurance exercise vs. no‐intervention control *The ADF groups' intervention included 4 weeks of controlled feeding followed by 8 weeks self‐selected feeding. ADF consisted of consuming 25% baseline energy needs on “fast” days alternated by “feed” days of ad libitum food intake*. *Control group was asked to maintain regular food and activity habits*.	**Setting:** free‐living **Outcomes:** **‐** Eating behavior traits (TFEQ‐R18) **‐** Self‐reported food intake (3‐day food record)	No follow‐up; just baseline and postintervention measurements
Brandon and Elliot‐Loyd (2006)	RCT	Intervention group 1 (African American exercisers): *N* = 15 Age: 34 (7) years BMI: 34.4 (8.2) kg/m^2^ % female: 100% Comorbidities: none Intervention group 2 (White exercisers): *N* = 13 Age: 41 (7) years BMI: 29.5 (5.7) kg/m^2^ % female: 100% Comorbidities: none Control group 1 (African American controls): *N* = 12 Age: 36 (4) years BMI: 33.0 (7.1) kg/m^2^ % female: 100% Comorbidities: none Control group 2 (White controls): *N* = 12 Age: 42 (10) years BMI: 32.8 (7.3) kg/m^2^ % female: 100% Comorbidities: none	**‐ Program duration:** 16 weeks (after 2‐week training session) **‐Number of sessions/week:** 3 days/week **‐ Type of training:** brisk walking (self‐paced, goal ~5.6 km/h), 4.8 km/session **‐ Supervision:** yes, unclear by whom	African American exercisers vs. White exercisers vs. African American controls vs. White controls	**Setting:** free‐living **Outcomes:** **‐** Self‐reported food intake (2‐day food record on a Sunday and Monday)	No follow‐up; just week 1, 9 and postintervention measurements
Di Blasio et al. (2010)	Non‐RTC	Intervention group 1 (morning exercise): *N* = 14 Age: 52 (3) years BMI: ≥25 kg/m^2^ % female: 100% (postmenopausal) Comorbidities: none Intervention group 2 (evening exercise): *N* = 15 Age: 53 (3) years BMI: ≥25 kg/m^2^ % female: 100% (postmenopausal) Comorbidities: none	**‐Program duration:** 3 months **‐Number of sessions/week:** 4 days/week **‐ Type of training:** walking 50 min at 55% heart rate reserve Morning exercise: 7‐9 a.m. (after breakfast) Evening exercise: 6‐8 p.m. (before dinner) **‐ Supervision:** partial; 2/4 sessions supervised by exercise trainer	Morning vs. evening walking (self‐selected)	**Setting:** free‐living **Outcomes:** **‐** Self‐reported food intake (3‐day dietary records)	No follow‐up; just baseline and postintervention measurements
Donnelly et al. (2003)	RCT	Intervention group: *N* = 41 Age: 17–35 years BMI males 29.7 (2.9) kg/m^2^ BMI females: 28.7 (3.2) kg/m^2^ % female: 61% Comorbidities: none Control group: *N* = 33 Age: 17–35 years BMI males: 29.0 (3.0) kg/m^2^ BMI females: 29.3 (2.3) kg/m^2^ % female: 55% Comorbidities: none	**‐Program duration:** 16 months **‐Number of sessions/week:** 5 days/week **‐ Type of training:** treadmill walking (1/5 days cycling and elliptical allowed) starting at 20 min at 60% heart rate reserve to 45 min at 75% heart rate reserve (~400 kcal per session, ~2000 kcal/week) **‐ Supervision:** yes, research personnel	Exercise vs. no‐exercise control *Control group instructed to maintain normal physical activity and dietary patterns*	**Setting:** university cafeteria and free‐living **Outcomes:** **‐** Measured food intake (2 weeks at baseline, 3, 6, 9, 12, and 16 months using digital photography in the cafeteria; food intake outside cafeteria assessed via 24‐h recall)	No follow‐up; just baseline, 3, 6, 9, 12 months, and postintervention measurements
Dorling et al. (2019)	Secondary analysis of an RCT	African American (intervention and control groups combined): *N* = 53 Age: 46 (10) years BMI: 33.4 (4.8) kg/m^2^ % female: NR Comorbidities: none White (intervention and control groups combined): *N* = 111 Age: 50 (12) years BMI: 30.6 (4.2) kg/m^2^ % female: NR Comorbidities: none	**‐Program duration:** 24 weeks **‐ Number of sessions/week:** 3–5 days/week (self‐selected) **‐ Type of training:** treadmill exercise (65%–85% VO_2peak_): 8 kcal/kg body weight/week (8 KKW; ~700 kcal/week) vs. 20 KKW (~1760 kcal/week) **‐ Supervision:** yes; unclear by whom	8 KKW vs. 20 KKW vs. no‐exercise control *Control group instructed to maintain baseline level of physical activity but received multimedia health information twice weekly and monthly seminars on healthy lifestyle*.	**Setting:** laboratory **Outcomes:** **‐** Measured energy intake (ad libitum lunch and dinner test meals) **‐** Hunger, fullness, desire to eat, prospective food consumption and satisfaction (VAS before after test meals) **‐** Satiety quotient	No follow‐up; just baseline and postintervention measurements
Flack et al. (2018)	Randomized trial	Intervention group 1 (1500 kcal/week): *N* = 18 Age: 27 (6) years BMI: 30.7 (4.3) kg/m^2^ % female: 72% Comorbidities: none Intervention group 2 (3000 kcal/week): *N* = 18 Age: 29 (5) years BMI: 29.6 (3.0) kg/m^2^ % female: 67% Comorbidities: none	**‐Program duration:** 12 weeks **‐Number of sessions/week:** 5 days/week **‐ Type of training:** 300 or 600 kcal/session (1500 kcal/week or 3000 kcal/week), 2 days/week lower intensity session (45%–64% heart rate reserve) and 3 days/week interval‐based sessions (65%–85% heart rate reserve) **‐ Supervision:** no, but activity tracker worn for each session and compliance monitored.	1500 vs. 3000 kcal/week	**Setting:** laboratory and free‐living **Outcomes:** **‐** Self‐reported food intake (3 days of Automated Self‐Administered 24‐h Dietary Recall) **‐** Food reinforcement task (2–4 h postprandial)	No follow‐up; just baseline and postintervention measurements
Foster‐Schubert et al. (2012)	RCT	Intervention group 1 (dietary weight loss): *N* = 118 Age: 58 (6) years BMI: 31.1 (3.9) kg/m^2^ % female: 100% (postmenopausal) Comorbidities: none Intervention group 2 (aerobic exercise): *N* = 117 Age: 58 (5) years BMI: 30.7 (3.7) kg/m^2^ % female: 100% (postmenopausal) Comorbidities: none Intervention group 3 (diet + exercise): *N* = 117 Age: 58 (5) years BMI: 31.0 (4.3) kg/m^2^ % female: 100% (postmenopausal) Comorbidities: none Control group: *N* = 87 Age: 58 (5) years BMI: 30.7 (3.9) kg/m^2^ % female: 100% (postmenopausal) Comorbidities: none	**‐Program duration:** 12 months **‐Number of sessions/week:** 5 days/week **‐ Type of training:** moderate‐to‐vigorous aerobic exercise, ≥45 min at 70%–85% maximal heart rate (progressing from 15 min at 60%–70% maximal heart rate) **‐ Supervision:** partial, ≥3/5 sessions supervised by exercise physiologist	Exercise vs. diet vs. diet + exercise vs. no‐intervention control *Diet group goals were “total daily energy intake of 1,200–2,000 kcal/day based on baseline weight, <30% daily energy intake from fat, and a 10% reduction in body weight by 6 months with maintenance thereafter to 12 months.”* *Control group requested not to change diet or exercise habits*	**Setting:** free‐living **Outcomes:** **‐** Self‐reported food intake (food frequency questionnaire)	No follow‐up; just baseline and postintervention measurements
Guelfi et al. (2013)	Unspecified	Intervention group 1 (aerobic training): *N* = 12 Age: 49 (7) years (groups combined) BMI: 31.7 (3.5) kg/m^2^ % female: 0% Comorbidities: none Intervention group 2 (resistance training): *N* = 13 Age: 49 (7) years (groups combined) BMI: 30.3 (3.5) kg/m^2^ % female: 0% Comorbidities: none Control group: *N* = 8 Age: 49 (7) years (groups combined) BMI: 30.1 (6.3) kg/m^2^ % female: 0% Comorbidities: none	**‐Program duration:** 12 weeks **‐Number of sessions/week:** 3 days/week **‐ Type of training:** aerobic exercise (40–60 min at 70%–80% HR_max_) or resistance exercise (weight training matched for duration and intensity; 3–4 sets 8–10 repetitions of 9 exercises at 75%–85% 1 repetition maximum) **‐ Supervision:** yes, unclear by whom	Aerobic training vs. resistance training vs. no‐exercise control *Control group asked to continue normal sedentary routine*.	**Setting:** laboratory (2‐h, 75‐g oral glucose tolerance test) **Outcomes:** **‐** Hunger and fullness (VAS)	No follow‐up; just baseline and postintervention measurements
Halliday et al. (2017)	Randomized trial	Intervention group: *N* = 170 Age: 60 (6) years BMI: 32.9 (3.8) kg/m^2^ % female: 73% Comorbidities: prediabetes *Participants randomized to one of two intervention maintenance groups after the 12‐week supervised intervention: social cognitive theory‐based or standard usual care*	**‐ Program duration:** 15 months (12 weeks supervised intervention followed by 6‐month intervention maintenance phase and 6‐month no‐contact phase) **‐Number of sessions/week:** 2 days/week **‐ Type of training:** resistance exercise, whole‐body routine targeting major muscle groups, with twelve exercises per session (one set of each exercise to concentric failure; ~35–45 min/session) **‐ Supervision:** partial, by personal trainers during first 12 weeks	None (data pooled)	**Setting:** free‐living **Outcomes:** **‐** Self‐reported food intake (average of three 24‐h recalls) **‐** Diet quality (HEI‐2010)	6 months; measurements done at baseline, 3 (postintervention), 9 and 15 months.
Heiston et al. (2019)	Randomized trial	Intervention group 1 (continuous exercise training): *N* = 14 Age: 62 (2) years BMI: 34.5 (7.1) kg/m^2^ % female: 79% Comorbidities: prediabetes Intervention group 2 (high‐intensity interval exercise; HIIT): *N* = 14 Age: 60 (2) years BMI: 32.1 (4.7) kg/m^2^ % female: 79% Comorbidities: prediabetes	**‐Program duration:** 2 weeks **‐Number of sessions/week:** daily (12 sessions) **‐ Type of training:** Continuous: 60 min at 70% heart rate peak HIIT: 60 min of alternating 3‐min intervals at 90%/50% heart rate peak **‐ Supervision:** yes, unclear by whom	Continuous vs. high‐intensity interval exercise	**Setting:** laboratory and free‐living **Outcomes:** **‐** Self‐reported food intake (3‐day food records) **‐** Hunger and fullness (VAS) in response to a 75‐g oral glucose tolerance test	No follow‐up; just baseline and postintervention measurements
Holliday et al. (2018)	RCT	Groups combined: *N* = 76 Age: 41 (2) years BMI: 29.2 (3.4) kg/m^2^ % female: 100% Comorbidities: none	**‐Program duration:** 24 weeks **‐Number of sessions/week:** ~5 days/week **‐ Type of training:** Points‐based exercise: points (derived from MET scores) allocated per 10 min of activity, 30 points/week (equating to 5 × 30 min brisk walking/week). Activities accumulated in bouts ≥10 min. Structured exercise: 5 × 30 min/week of moderate intensity exercise **‐ Supervision:** none; participants contacted twice weekly for first 4 weeks, then every 2 weeks from week 4–12 and no contact from week 13–24.	Points‐based physical activity vs. structure exercise vs. waiting‐list control *Control group instructed to maintain current lifestyle*.	**Setting:** free‐living **Outcomes:** **‐** Self‐reported food intake (3‐day weighed food record; *n* = 41)	No follow‐up; just baseline and postintervention measurements
Jakicic et al. (2011)	RCT	Intervention group 1 (moderate dose): *N* = 76 Age: 44 (8) years BMI: 27.2 (1.8) kg/m^2^ % female: 91% Comorbidities: none Intervention group 2 (high dose): *N* = 88 Age: 46 (8) years BMI: 27.0 (1.6) kg/m^2^ % female: 92% Comorbidities: none Control group (self‐help): *N* = 84 Age: 45 (8) years BMI: 27.1 (1.7) kg/m^2^ % female: 92% Comorbidities: none *Secondary analyses performed on those who maintained weight (±3%, n* = *132), gained (n = 48) or lost (n = 68) weight (>3%) after 18 months*.	**‐Program duration:** 18 months **‐Number of sessions/week:** >5 days/week **‐ Type of training:** moderate‐dose (150 min/week), high‐dose (300 min/week) in bouts ≥10 min moderate‐to‐vigorous intensity (55%–85% HR_max_) **‐Supervision:** none	Moderate dose vs. high dose vs. self‐help control group *Self‐help group received a physical activity self‐help manual*	**Setting:** free‐living **Outcomes:** **‐** Self‐reported food intake (food frequency questionnaire) **‐** Eating Behavior Inventory	No follow‐up; just baseline, 6 months, 12 months and postintervention measurements
Kirkwood et al. (2007)	RCT	Intervention group 1 (energy‐reduced diet): *N* = 16 Age: 30–50 years BMI: 30.1 (4.1) kg/m^2^ % female: 100% Comorbidities: none Intervention group 2 (activity): *N* = 19 Age: 30–50 years BMI: 31.6 (3.8) kg/m^2^ % female: 100% Comorbidities: none Intervention group 3 (diet + activity): *N* = 16 Age: 30–50 years BMI: 32.2 (4.6) kg/m^2^ % female: 100% Comorbidities: none Control group: *N* = 18 Age: 30–50 years BMI: 32.5 (4.5) kg/m^2^ % female: 100% Comorbidities: none	**‐Program duration:** 12 weeks **‐Number of sessions/week:** daily **‐ Type of training:** 60 min of brisk walking **‐Supervision:** none	Diet vs. activity vs. diet + activity vs. no‐intervention control *Diet group received “specific advice recommending a high‐carbohydrate (50%–55% energy, of which 10% is sucrose), low‐fat diet (30%–35% energy) as detailed in the ‘System S’ Plan.”* *Control group received no advice*	**Setting:** free‐living **Outcomes:** **‐** Self‐reported food intake (4‐day unweighed food diary)	No follow‐up; just baseline, week 6 and postintervention measurements.
Macias‐Cervantes et al. (2015)	Randomized trial	Intervention group 1 (low advanced glycation end product diet): *N* = 14 Age: 40 (5) years BMI: 29.4 (2.2) kg/m^2^ % female: 0% Comorbidities: none Intervention group 2 (exercise): *N* = 14 Age: 44 (7) years BMI: 28.3 (1.7) kg/m^2^ % female: 0% Comorbidities: none Intervention group 3 (diet + exercise): *N* = 15 Age: 44 (5) years BMI: 28.9 (2.2) kg/m^2^ % female: 0% Comorbidities: none	**‐Program duration:** 12 weeks **‐Number of sessions/week:** 3 days/week **‐ Type of training:** aerobic exercise (running) 45 min at 65%–75% maximal heart rate **‐ Supervision:** yes, researcher from municipal sport center	Low advance glycation end product diet (energetic) vs. exercise vs. diet + exercise *Diet groups “were given precise instructions on how to follow a diet that maintained their caloric and nutrient intakes but significantly reduced [advance glycation end product] content; the latter was achieved mostly by changing cooking methods in food preparation to avoid exposure to dry heat such as frying, broiling, grilling, and roasting and to favor cooking with lower temperatures and high‐water, content as in stewing and poaching.”*	**Setting:** free‐living **Outcomes:** **‐** Self‐reported food intake (method NR)	No follow‐up; just baseline and postintervention measurements
Martin et al. (2019)	RCT	Intervention group 1 (8 kcal/kg/week [KKW]): *N* = 59 Age: 48 (11) years BMI: 31.4 (4.6) kg/m^2^ % female: 73% Comorbidities: none Intervention group 2 (20 KKW): *N* = 51 Age: 49 (12) years BMI: 30.6 (4.4) kg/m^2^ % female: 71% Comorbidities: none Control group: *N* = 61 Age: 50 (11) years BMI: 32.3 (4.8) kg/m^2^ % female: 74% Comorbidities: none *Pooled exercisers (n = 110) divided into compensators (C)/noncompensators (NC) based on actual and predicted weight loss (median split)*.	**‐Program duration:** 24 weeks **‐ Number of sessions/**week**:** 3–5 days/week (self‐selected) **‐ Type of training:** treadmill exercise (65%–85% VO_2peak_): 8 kcal/kg body weight/week (8 KKW; ~700 kcal/week) vs. 20 KKW (~1760 kcal/week) **‐ Supervision:** yes; unclear by whom	8 KKW vs. 20 KKW vs. no‐exercise control *Control group instructed to maintain baseline level of physical activity but received multimedia health information twice weekly and monthly seminars on healthy lifestyle*.	**Setting:** laboratory **Outcomes:** **‐** Energy intake (inferred from doubly labeled water [DLW] and via ad libitum lunch and dinner test meals) **‐** Hunger, fullness, desire to eat, prospective food consumption and satisfaction (VAS before after test meals and retrospectively over previous week) **‐** Eating behavior traits (Eating Inventory/TFEQ, Food Preference Questionnaire, Food Craving Inventory, Yale Food Addiction Scale)	No follow‐up; just baseline and postintervention measurements
Martins et al. (2017)	Randomized trial	Intervention group 1 (high‐intensity interval training; HIIT): *N* = 13 completers Age: 34 (8) years BMI: 33.2 (3.5) kg/m^2^ % female: 60% Comorbidities: none Intervention group 2 (½ high‐intensity interval training; ½‐HIIT: *N* = 9 completers Age: 34 (7) years BMI: 32.4 (2.9) kg/m^2^ % female: 20% Comorbidities: none Intervention group 3 (moderate intensity continuous training; MICT): *N* = 13 completers Age: 33 (10) years BMI: 33.3 (2.4) kg/m^2^ % female: 40% Comorbidities: none	**‐Program duration:** 12 weeks **‐Number of sessions/week:** 3 days/week **‐ Type of training:** HIIT (8 s at 85%–90% HR_max_ and 12 s recovery for 250 kcal), ½‐HIIT (8 s at 85%–90% HR_max_ and 12 s recovery for 125 kcal), and MICT (250 kcal at 70% of HR_max_) **‐ Supervision:** yes; unclear by whom	HIIT vs. ½‐HIIT vs. MICT	**Setting:** laboratory **Outcomes:** **‐** Hunger, fullness, prospective food consumption and desire to eat (VAS) over 3 h postbreakfast (600 kcal) **‐** Food reward (LFPQ) before and after breakfast	No follow‐up; just baseline and postintervention measurements
Nieman et al. (1990)	RCT	Intervention group: *N* = 18 Age: 36 (7) years BMI: 28.3 (3.0) kg/m^2^ % female: 100% (premenopausal) Comorbidities: none Control group: *N* = 18 Age: 33 (6) years BMI: 27.8 (3.8) kg/m^2^ % female: 100% (premenopausal) Comorbidities: none	**‐Program duration:** 15 week **‐Number of sessions/week:** 5 days/week **‐ Type of training:** walking on measured course for 45 min at 60% heart rate reserve **‐ Supervision:** yes; by “supervisor”	Exercise vs. no‐exercise control *Control group instructed not to participate in any exercise outside of normal daily activity*.	**Setting:** free‐living **Outcomes:** **‐** Self‐reported food intake (7‐day food records)	No follow‐up; just baseline, week 6 and postintervention measurements.
Quist et al. (2019)	RCT	Intervention group 1 (bike): *N* = 22 Age: 35 (7) years BMI: 30.1 (3.3) kg/m^2^ % female: 55% Comorbidities: none Intervention group 2 (moderate‐intensity exercise): *N* = 33 Age: 33 (7) years BMI: 29.2 (1.9) kg/m^2^ % female: 48% Comorbidities: none Intervention group 3 (vigorous‐intensity exercise): *N* = 25 Age: 37 (7) years BMI: 30.0 (2.4) kg/m^2^ % female: 52% Comorbidities: none Control group: *N* = 16 Age: 35 (7) BMI: 30.1 (2.3) kg/m^2^ % female: 44% Comorbidities: none	**‐Program duration:** 6 months **‐Number of sessions/week:** 5 days/week **‐ Type of training:** 320 kcal/d for women and 420 kcal/d for men of active commuting by bike, or moderate‐intensity (50% VO_2peak_) or vigorous‐intensity (70% VO_2peak_) aerobic leisure‐time exercise **‐ Supervision:** none, but heart rate monitor worn for all exercise sessions	Active commuting by bike vs. moderate‐intensity exercise vs. vigorous‐intensity exercise vs. no‐exercise control	**Setting:** laboratory and free‐living (standardized breakfast and snack followed by exercise challenge and ad libitum lunch at baseline, 3 months and 6 months) **Outcomes:** **‐** Energy intake (ad libitum lunch meal and food record remainder of the day) **‐** Hunger, satiety, fullness, prospective food consumption (VAS) throughout test day over 315 min **‐** Restraint, disinhibition, susceptibility to hunger (TFEQ)	No follow‐up; just baseline, 3 months and postintervention measurements
Reseland et al. (2001)	RCT	Intervention group 1 (diet): *N* = 44 Age: 45 (3) years (groups combined) BMI: 27.8 (3.5) kg/m^2^ % female: 0% Comorbidities: metabolic syndrome Intervention group 2 (exercise): *N* = 48 Age: 45 (3) years (groups combined) BMI: 28.2 (3.3) kg/m^2^ % female: 0% Comorbidities: metabolic syndrome Intervention group 3 (diet + exercise): *N* = 57 Age: 45 (3) years (groups combined) BMI: 26.2 (2.6) kg/m^2^ % female: 0% Comorbidities: metabolic syndrome Control group: *N* = 37 Age: 45 (3) years (groups combined) BMI: 28.8 (3.4) kg/m^2^ % female: 0% Comorbidities: metabolic syndrome	**‐Program duration:** 1 year **‐Number of sessions/week:** 3 days/week **‐ Type of training:** endurance exercise (aerobics, circuit training, fast walking/jogging) for 60 min at 60%–80% peak heart rate **‐ Supervision:** yes (supervised groups)	Diet vs. exercise vs. diet + exercise vs. no‐intervention control *Diet groups received counseling at baseline, 3 months and 9 months. “The advice was individually tailored according to dietary habits and risk factor profile. Increased consumption of fish and fish products, vegetables, and fiber‐rich products containing complex carbohydrates and reduced intake of saturated fat and cholesterol were recommended.”*	**Setting:** free‐living **Outcomes:** **‐** Self‐reported food intake (food frequency questionnaire)	No follow‐up; just baseline and postintervention measurements
Rhew et al. (2007)	RCT	Intervention group: *N* = 87 Age: 61 (7) years BMI: 30.4 (4.1) kg/m^2^ % female: 100% (postmenopausal) Comorbidities: none Control group: *N* = 86 Age: 61 (7) years BMI: 30.5 (3.7) kg/m^2^ % female: 100% (postmenopausal) Comorbidities: none	**‐Program duration:** 12 months **‐Number of sessions/week:** 5 days/week **‐ Type of training:** aerobic exercise from 16 min at 40% VO_2max_ building up to 45 min at 60%–70% VO_2max_ **‐ Supervision:** partial; first 3 months 3/5 sessions/week at facility, months 4–12 ≥ 1 session/week at facility.	Exercise vs. control (stretching/relaxation) *Control group attended weekly 60‐mins stretching/relaxation sessions for the entire year and asked not to change other exercise habits*.	**Setting:** free‐living **Outcomes:** **‐** Self‐reported food intake (food frequency questionnaire; analyses restricted to those reporting 600–4000 kcal/day)	No follow‐up; just baseline, month 3 and postintervention measurements.
Riou et al. (2019)	Randomized trial	Intervention group 1 (low‐intensity): *N* = 11 Age: 27 (9) years BMI: 32.3 (3.8) kg/m^2^ % female: 100% (premenopausal) Comorbidities: none Intervention group 2 (moderate‐intensity): *N* = 10 Age: 31 (11) years BMI: 35.1 (6.2) kg/m^2^ % female: 100% (premenopausal) Comorbidities: none	**‐Program duration:** 12 weeks **‐Number of sessions/week:** 5 days/week **‐ Type of training:** aerobic exercise (300 kcal/d) at low (40% VO_2reserve_) or moderate (60% VO_2reserve_) intensity **‐ Supervision:** partial; 3 days/week supervised in the laboratory (participants wore a heart rate monitor for all sessions)	Low‐intensity vs. moderate‐intensity	**Setting:** laboratory and free‐living Self‐selected fixed breakfast followed by exercise challenge and ad libitum test meal **Outcomes:** **‐** Energy intake (ad libitum food menu for 1.5 days and 7‐day food record) **‐** Hunger, fullness, desire to eat and prospective food consumption (VAS) before and after test meals **‐** Eating behavior traits (TFEQ) ‐ Food reward (Leeds Food Preference Questionnaire)	No follow‐up; just baseline (week 4), week 1 and postintervention measurements
Rosenkilde et al. (2012)	RCT	Intervention group 1 (moderate‐dose): *N* = 18 Age: 30 (7) years BMI: 28.6 (1.8) kg/m^2^ % female: 0% Comorbidities: none Intervention group 2 (high‐dose): *N* = 18 Age: 28 (5) years BMI: 27.6 (1.4) kg/m^2^ % female: 0% Comorbidities: none Control group: *N* = 17 Age: 31 (6) years BMI: 28.0 (2.3) kg/m^2^ % female: 0% Comorbidities: none	**‐Program duration:** 13 weeks ‐ **Number of sessions/week:** daily **‐ Type of training:** endurance exercise expending 300 kcal/day (moderate‐dose) or 600 kcal/day (high‐dose) at >70% VO_2max_ 3 days/week and self‐selected intensity on other days. **‐ Supervision:** none (heart rate monitor worn during all exercise sessions)	Moderate‐dose vs. high‐dose vs. sedentary control	**Setting:** free‐living **Outcomes:** **‐** Self‐reported food intake (3‐day weighed food records) at baseline and week 11 **‐** Measured high‐carbohydrate or low‐carbohydrate ad libitum food intake (4 days each condition) at baseline and week 13	No follow‐up; just baseline and postintervention measurements
Rosenkilde et al. (2013)	RCT	Intervention group 1 (moderate‐dose): *N* = 18 Age: 30 (7) years BMI: 28.6 (1.8) kg/m^2^ % female: 0% Comorbidities: none Intervention group 2 (high‐dose): *N* = 18 Age: 28 (5) years BMI: 27.6 (1.4) kg/m^2^ % female: 0% Comorbidities: none Control group: *N* = 17 Age: 31 (6) years BMI: 28.0 (2.3) kg/m^2^ % female: 0% Comorbidities: none	**‐Program duration:** 12 weeks **‐Number of sessions/week:** daily **‐ Type of training:** endurance exercise expending 300 kcal/day (moderate‐dose) or 600 kcal/day (high‐dose) at >70% VO_2max_ 3 days/week and self‐selected intensity on other days. **‐ Supervision:** none (heart rate monitor worn during all exercise sessions)	Moderate‐dose vs. high‐dose vs. sedentary control	**Setting**: laboratory Test day 1: appetite response to standardized breakfast Test day 2: appetite response to acute exercise (1 h ~ 60% VO_2max_) **Outcomes:** **‐** Hunger, satiety, fullness, prospective food consumption, palatability and liking (VAS) **‐** Measured food intake (lunch test meal after standardized breakfast of 600 kcal) **‐** Restraint, disinhibition and susceptibility to hunger (TFEQ)	No follow‐up; just baseline and postintervention measurements
Sim et al. (2015)	RCT	Intervention group 1 (high‐intensity interval training; HIIT): *N* = 10 Age: 31 (8) years (groups combined) BMI: 27.4 (1.6) kg/m^2^ % female: 0% Comorbidities: none Intervention group 2 (moderate‐intensity continuous training; MICT): *N* = 10 Age: 31 (8) years (groups combined) BMI: 27.2 (1.5) kg/m^2^ % female: 0% Comorbidities: none Control group: *N* = 10 Age: 31 (8) years (groups combined) BMI: 27.0 (0.9) kg/m^2^ % female: 0% Comorbidities: none	**‐Program duration:** 12 weeks **‐Number of sessions/week:** 3 days/week **‐ Type of training:** cycling HIIT (15 s at 170% VO_2peak_ and 60 s at 32% VO_2peak_) or continuous exercise (60% VO_2peak_) starting with 30 min and increasing by 5 min every 3 weeks to 45 min. **‐ Supervision:** yes; by researcher	HIIT vs. MICT vs. no‐exercise control	**Setting:** laboratory and free‐living. Low‐energy (~200 kcal) and high‐energy (~580 kcal) preload test days. **Outcomes:** **‐** Hunger, fullness, satiation, desire to eat, and prospective food consumption (VAS) **‐** Energy intake (1 test meal after preload and food record for remainder of the day)	No follow‐up; just baseline and postintervention measurements
Washburn et al. (2012)	RCT	Intervention group: *N* = 32 Age: 20 (2) years BMI: 27.5 (2.9) kg/m^2^ % female: 47% Comorbidities: none Control group: *N* = 23 Age: 21 (3) years BMI: 27.1 (2.8) kg/m^2^ % female: 43% Comorbidities: none	**‐Program duration:** 6 months **‐Number of sessions/week:** 3 days/week **‐ Type of training:** resistance training, 1 set, 9 exercises, 3–6 repetition maximum (~11 min) **‐ Supervision:** yes; by laboratory technician	Resistance exercise vs. no‐exercise control	**Setting:** free‐living **Outcomes:** **‐** Self‐reported food intake (monthly by 24‐h recalls for 2 weekdays and 1 weekend day).	No follow‐up; just baseline, monthly and postintervention measurements
Washburn et al. (2015)	RCT	Intervention group 1 (400 kcal/session): *N* = 36 Age: 23 (3) years BMI: 31.2 (5.6) kg/m^2^ % female: 50% Comorbidities: none Intervention group 2 (600 kcal/session): *N* = 37 Age: 23 (4) years BMI: 30.6 (3.9) kg/m^2^ % female: 49% Comorbidities: none Control group: *N* = 18 Age: 23 (3) years BMI: 29.7 (3.8) kg/m^2^ % female: 50% Comorbidities: none	**‐Program duration:** 10 months **‐Number of sessions/week:** 5 days/week **‐ Type of training:** walking/jogging with 1 session/week alternative activities (e.g., stationary biking, walking/jogging outside or elliptical) building up to 400 kcal/session or 600 kcal/session at 70%–80% HR_max._ **‐ Supervision:** yes; by trained research staff	400 kcal/session vs. 600 kcal/session vs. no‐exercise control *Control group asked to continue typical patterns of physical activity and dietary intake*.	**Setting:** university cafeteria and free‐living **Outcomes:** **‐** Measured food intake (7 days at baseline, 3.5, 7, and 10 months using digital photography in the cafeteria; food intake outside cafeteria assessed via 24‐h recall) **‐** Diet quality (Healthy Eating Index 2010)	No follow‐up; just baseline and month 3.5, 7 and postintervention measurements
Willis et al. (2019)	Secondary analyses of an RCT	Intervention group 1 (Early Exercise): *N* = 21 Age: 24 (4) years BMI: 29.7 (3.6) kg/m^2^ % female: 48% Comorbidities: none Intervention group 2 (Late Exercise): *N* = 25 Age: 24 (3) years BMI: 32.0 (5.5) kg/m^2^ % female: 44% Comorbidities: none Intervention group 3 (Sporadic Exercise): *N* = 24 Age: 21 (2.3) years BMI: 30.6 (4.9) kg/m^2^ % female: 63% Comorbidities: none Control group: *N* = 18 Age: 23 (3) years BMI: 29.5 (3.6) kg/m^2^ % female: 50% Comorbidities: none	**‐Program duration:** 10 months **‐Number of sessions/week:** 5 days/week **‐ Type of training:** walking/jogging with 1 session/week alternative activities (e.g., stationary biking, walking/jogging outside, or elliptical) building up to 400 kcal/session or 600 kcal/session at 70%–80% HR_max._ **‐ Supervision:** yes; by trained research staff *Participants retrospectively categorized into Early Exercise (>50% exercise between 7:00–11:59 a.m.), Late Exercise (>50% exercise between 3:00–7:00 p.m.) or Sporadic Exercise (<50% exercise any time)*.	Early vs. late vs. sporadic exercise vs. no‐exercise control *Control group asked to continue typical patterns of physical activity and dietary intake*	**Setting:** university cafeteria and free‐living **Outcomes:** **‐** Measured food intake (7 days at baseline, 3.5, 7, and 10 months using digital photography in the cafeteria; food intake outside cafeteria assessed via 24‐h recall)	No follow‐up; just baseline and month 3.5, 7 and postintervention measurements
**Single‐group interventions**						
Bryant et al. (2012)	Single group intervention	*N* = 58 Age: 36 (10) years BMI: 31.8 (4.5) kg/m^2^ % female: 67% Comorbidities: none	**‐Program duration:** 12 weeks **‐Number of sessions/week:** 5 days/week **‐ Type of training:** aerobic exercise (500 kcal at 70% HR_max_) **‐ Supervision:** yes; research staff *Participants retrospectively classified into responders (n = 32) or nonresponders (n = 26) based on actual weight change compared to that predicted from the changes in body composition*.	Responders vs. nonresponders	**Setting**: laboratory **Outcomes:** **‐** Measured food intake (self‐determined fixed breakfast followed by ad libitum lunch, dinner and evening snack box) **‐** Restraint, disinhibition and susceptibility to hunger (TFEQ)	No follow‐up; just baseline, week 4, week 8 and postintervention measurements
Caudwell et al. (2013a)	Single group intervention	*N* = 41 Age: 43 (8) years BMI: 30.7 (3.9) kg/m^2^ % female: 66% (premenopausal) Comorbidities: none	**‐Program duration:** 12 weeks **‐Number of sessions/week:** 5 days/week **‐ Type of training:** aerobic exercise (500 kcal at 70% HR_max_) **‐ Supervision:** yes; research staff	None	**Setting**: laboratory High‐energy and low‐energy density probe days **Outcomes:** **‐** Measured food intake (self‐determined fixed breakfast, fixed energy lunch and ad libitum dinner and evening snack box)	No follow‐up; just baseline and postintervention measurements
Caudwell et al. (2013b)	Single group intervention	Males *N* = 35 Age: 41 (9) years BMI: 30.5 (8.6) kg/m^2^ Comorbidities: none Females (premenopausal) *N* = 72 Age: 41 (10) years BMI: 31.8 (4.3) kg/m^2^ Comorbidities: none	**‐Program duration:** 12 weeks **‐Number of sessions/week:** 5 days/week **‐ Type of training:** aerobic exercise (500 kcal at 70% HR_max_) **‐ Supervision:** yes; research staff	Males vs. females	**Setting**: laboratory **Outcomes:** **‐** Hunger, fullness and desire to eat (VAS) **‐** Satiety quotient (SQ) **‐** Measured food intake (self‐determined fixed breakfast, fixed energy lunch and ad libitum dinner and evening snack box)	No follow‐up; just baseline and postintervention measurements
Cornier et al. (2012)	Single group intervention	*N* = 12 Age: 38 (10) years BMI: 33.3 (4.3) kg/m^2^ % female: 58% Comorbidities: none	**‐Program duration:** 6 months **‐Number of sessions/week:** 5 days/week **‐ Type of training:** treadmill walking 5 (building up to 500 kcal/d at 75% VO_2max_) **‐ Supervision:** yes; unclear by whom	None	**Setting**: laboratory and free‐living Test meal breakfast (30% estimated daily energy needs) **Outcomes:** **‐** Eating behavior traits (TFEQ, Power of Food Scale, Craving and Mood Questionnaire, Food Craving Inventory) **‐** Neuronal response to food cues (in response to chronic exercise and chronic + acute exercise) **‐** Hunger, satiety and prospective food consumption (VAS) **‐** Self‐reported food intake (3‐day food record)	No follow‐up; just baseline and postintervention measurements
Crampes et al. (2003)	Single group intervention	*N* = 11 Age: 26 (3) years BMI: 27.7 (0.7) kg/m^2^ % female: 0% Comorbidities: none	**‐Program duration:** 4 months **‐ Number of sessions/week:** 5 days/week (3 days running, 2 days cycling) **‐ Type of training:** aerobic exercise (50%–85% VO_2max_), 60 min per session **‐ Supervision:** yes, physical exercise coach	None	**Setting:** free‐living **Outcomes:** **‐** Self‐reported food intake (3‐day food record)	No follow‐up; just baseline and postintervention measurements
Garnier et al. (2015)	Single group intervention	*N* = 156 Age: 60 (5) years BMI: 30.0 (5.0) kg/m^2^ % female: 100% (postmenopausal) Comorbidities: none	**‐Program duration:** 16 weeks **‐ Number of sessions/**week**:** 3 days/week (nonconsecutive) **‐ Type of training:** 45 min of walking at 60% heart rate reserve **‐ Supervision:** partial; 2 days/week supervised by trained exercise leader *Participants retrospectively grouped into tertiles of body weight or fat mass loss*.	None	**Setting**: free‐living **Outcomes:** **‐** Self‐reported food intake (3‐day food record)	No follow‐up; just baseline and postintervention measurements
Halliday et al. (2014)	Single group intervention	Intervention group: *N* = 134 Age: 60 (6) years BMI: 25–39.9 kg/m^2^ % female: 70% Comorbidities: prediabetes *Participants reporting energy intake <80% resting metabolic rate excluded from analysis (n = 25)*	**‐Program duration:** 12 weeks **‐Number of sessions/week:** 2 days/week **‐ Type of training:** resistance exercise, whole‐body routine targeting major muscle groups, with 12 exercises per session (one set of each exercise to concentric failure; ~35–45 min/session) **‐ Supervision:** yes, by personal trainers	None	**Setting:** free‐living **Outcomes:** **‐** Self‐reported food intake (average of three 24‐h recalls)	No follow‐up; just baseline and postintervention measurements
Kanaley et al. (2014)	Single group intervention	*N* = 13 Age: 41 (7) years BMI: 35.5 (4.0) % female: 85% (premenopausal) Comorbidities: none	**‐ Program duration:** 15 days of exercise over a 3‐week period **‐Number of sessions/week:** NR **‐ Type of training:** 60‐min walking at 70% VO_2peak_ **‐ Supervision:** yes; study personnel	None	**Setting:** laboratory 12‐h study day with 250‐kcal shake consumed every 2 h (6 meals in total) **Outcomes:** **‐** Hunger and fullness every 20 min (VAS)	No follow‐up; just baseline and postintervention measurements
King et al. (2008)	Single group intervention	Compensators: *N* = 18 Age: 38 (9) years BMI: 30.7 (2.9) kg/m^2^ % female: 76% Comorbidities: none Noncompensators: *N* = 17 Age: 40 (13) years BMI: 33.1 (4.7) kg/m^2^ % female: 66% Comorbidities: none	**‐Program duration:** 12 weeks **‐Number of sessions/week:** 5 days/week **‐ Type of training:** aerobic exercise (500 kcal at 70% HR_max_) **‐ Supervision:** yes; research staff *Participants retrospectively classified into compensators or noncompensators based on actual weight change compared to their predicted changes*.	Compensators vs. noncompensators	**Setting:** laboratory **Outcomes:** **‐** Hunger, fullness, prospective food consumption and desire to eat (VAS) **‐** Measured food intake (self‐determined fixed breakfast followed by ad libitum lunch, dinner and evening snack box)	No follow‐up; just baseline and postintervention measurements
King et al. (2009)	Single group intervention	*N* = 58 Age: 40 (10) years BMI: 31.8 (4.5) kg/m^2^ % female: 67% Comorbidities: none	**‐Program duration:** 12 weeks **‐Number of sessions/week:** 5 days/week **‐ Type of training:** aerobic exercise (500 kcal at 70% HR_max_) **‐ Supervision:** yes; research staff *Participants retrospectively classified into responders (n = 32) or nonresponders (n = 26) based on actual body composition change compared to their predicted changes*.	Responders vs. nonresponders	**Setting**: laboratory **Outcomes:** **‐** Hunger, fullness, prospective food consumption and desire to eat (VAS) **‐** Satiety quotient **‐** Measured food intake (self‐determined fixed breakfast followed by ad libitum lunch, dinner and evening snack box)	No follow‐up; just baseline and postintervention measurements
Manthou et al. (2010)	Single group intervention	*N* = 34 Age: 32 (8) years BMI: 29.3 (4.4) kg/m^2^ % female: 100% Comorbidities: none	**‐Program duration:** 8 weeks **‐ Number of sessions/week:** pattern A (*n* = 18): 2 days/week for 75 min pattern B (*n* = 16): 5 days/week for 30 min **‐ Type of training:** 150 min/week at heart rate 135–145 beats/min (72%–77% maximum heart rate) **‐ Supervision:** yes; by a researcher *Participants retrospectively classified into responders (n = 11) or nonresponders (n = 23) based on predicted fat loss with actual fat loss*.	Responders vs. nonresponders	**Setting:** free‐living **Outcomes:** **‐** Self‐reported food intake (7‐day weighed food diary)	No follow‐up; just baseline and postintervention measurements
Martins et al. (2010)	Single group intervention	*N* = 15 Age: 37 (8) years BMI: 31.3 (2.3) kg/m^2^ % female: 47% Comorbidities: none	**‐Program duration:** 12 weeks **‐Number of sessions/week:** 5 days/week **‐ Type of training:** aerobic exercise (500 kcal at 75% HR_max_) **‐ Supervision:** yes; unclear by whom	None	**Setting**: laboratory **Outcomes:** **‐** Hunger, fullness, prospective food consumption and desire to eat (VAS) over 3 h after a standardized breakfast of 600 kcal	No follow‐up; just baseline and postintervention measurements
Martins et al. (2013)	Single group intervention	*N* = 15 Age: 37 (8) years BMI: 31.3 (2.3) kg/m^2^ % female: 47% Comorbidities: none	**‐Program duration:** 12 weeks **‐Number of sessions/week:** 5 days/week **‐ Type of training:** aerobic exercise (500 kcal at 75% HR_max_) **‐ Supervision:** yes; unclear by whom	None	**Setting:** laboratory and free‐living Appetite response to: 1) Standardized breakfast (600 kcal) 2) Low‐energy preload (246 kcal) 3) High‐energy preload (607 kcal) **Outcomes:** **‐** Food intake (1 test meal after preload and food record for remainder of the day) **‐** Hunger, fullness, prospective food consumption and desire to eat (VAS) pre and post‐preload, and then at 20, 40, and 60 min.	No follow‐up; just baseline and postintervention measurements
Myers et al. (2019)	Single group intervention	*N* = 24 Age: 33 (12) years BMI: 27.9 (2.7) kg/m^2^ % female: 100% Comorbidities: none	**‐Program duration:** 12 weeks **‐Number of sessions/week:** 5 days/week **‐ Type of training:** aerobic exercise (500 kcal at 70% HR_max_) **‐ Supervision:** yes; research staff	None	**Setting:** laboratory **Outcomes:** **‐** Measured energy intake (fixed breakfast at 25% resting metabolic rate, followed by ad libitum lunch, dinner, evening snack box) **‐** Hunger, fullness, desire to eat and prospective food consumption (VAS)	No follow‐up; just baseline and postintervention measurements
Woo et al. (1982a)	Single group inpatient intervention	*N* = 3 Age: 30 (15) years BMI: 38.4 (4.1) kg/m^2^ % female: 100% Comorbidities: NR	**‐Program duration:** 57 days ‐ **Number of sessions/week:** daily **‐ Type of training:** treadmill walking at 2.5% grade (self‐determined speed; ~4.8 km/h) to expend 125% sedentary expenditure (~111 min/d) **‐ Supervision:** yes; hospital staff	None	**Setting:** hospital **Outcomes:** **‐** Measured daily food intake (covertly weighed platters at breakfast, lunch and supper as well as snacks in room)	No follow‐up; just daily and postintervention measurements
**Crossover trials**						
Alkahtani et al. (2014)	Crossover study (each training block was counterbalanced and separated by a 6‐week detraining washout)	*N* = 10 Age: 29 (4) years BMI: 30.7 (3.4) kg/m^2^ % female: 0% Comorbidities: none	**‐Program duration:** 4 weeks **‐Number of sessions/week:** 3 days/week **‐ Type of training:** moderate‐intensity interval training (MIIT; 30–45 min of 5‐min stages at ±20% workload at 45% VO_2peak_) vs. high‐intensity interval training (HIIT; 30–45 min of 30‐s 90% VO_2peak_ and 30‐s rest) **‐ Supervision:** yes, researcher	MIIT vs. HIIT	**Setting:** laboratory (test meal following 45‐min cycling at 45% VO_2max_ pre and post both training blocks; ΔMedium term‐Ex = ΔAcute‐Ex wk 4 − ΔAcute‐Ex wk 0) **Outcomes:** **‐** Hunger, desire to eat and fullness (VAS) **‐** Liking and wanting (LFPQ) **‐** Measured food intake (test meal)	No follow‐up; just baseline and postintervention measurements
Damour et al. (2019)	Pilot crossover study (each training block was randomized and not separated by a washout period)	*N* = 8 Age: 26 (22–29) years^a^ BMI: NR % female: 87.5% Comorbidities: none	**‐Program duration:** 4 weeks **‐Number of sessions/week:** twice daily **‐ Type of training:** 3‐min warm up (rating of perceived exertion [RPE]: 5–7/10), 6 1‐min high‐intensity intervals (RPE: 8–9/10) with 30‐sec rest (RPE 5–7/10), 3‐min cool down (RPE: 5–7/10)) performed 1 h before any two meals (ExMeal) or any other time (Ex) **‐Supervision:** none	Exercise performed 1 h before any two meals (ExMeal) vs. any other time (Ex)	**Setting:** free‐living **Outcomes:** **‐** Self‐reported food intake (web‐based food frequency questionnaire)	No follow‐up; just baseline and postintervention measurements
Woo et al. (1982b)	Latin square crossover design (three consecutive 19‐day treatment periods)	*N* = 6 Age: 43 (14) years BMI: 34.3 (3.6) kg/m^2^ % female: 100% Comorbidities: NR	**‐Program duration:** 19 days **‐Number of sessions/week:** daily **‐ Type of training:** treadmill walking at 2.5% grade (self‐determined speed; ~4.8 km/h) to expend 110% (mild exercise; ~39 min/d) or 125% (moderate exercise; ~96 min/d) of sedentary expenditure **‐Supervision:** NR	Sedentary (no exercise) vs. mild vs. moderate exercise	**Setting:** hospital **Outcomes:** **‐** Measured daily food intake (covertly weighed platters at breakfast, lunch and supper as well as snacks in room)	No follow‐up; just daily and postintervention measurements

*Note*: Values are means (SD), unless specified otherwise.

Abbreviations: ADF, alternate day fasting; AT, aerobic training; C, compensators; DLW, doubly labeled water; EX, exercise; ExMeal, exercise before meal; HIIT, high‐intensity interval exercise; HR, heart rate; KKW, kcal/kg body weight/week; LFPQ, Leeds Food Preference Questionnaire; MICT, moderate‐intensity continuous training; MIIT, moderate‐intensity interval training; NC; noncompensators; NR, not reported; RPE, ratings of perceived exertion; RT, resistance training; TFEQ, Three‐Factor Eating Questionnaire; VAS, visual analogue scale.

^a^ Median (interquartile range/25th, 75th quartile).

The studies were published between 1982 and 2020. The studies included randomized (*n* = 25) or nonrandomized (*n* = 5) trials, single‐group intervention studies (*n* = 15), and cross‐over trials (*n* = 3). Twenty studies included a no‐exercise control group, six compared different exercise modalities (e.g., aerobic, resistance, combined aerobic and resistance, and/or HIIT), eight compared different exercise doses/intensities, four compared different exercise timing conditions (relative to a meal or diurnal timing), two compared different races (White vs. African American), and four compared noncompensators/responders versus compensators/non‐responders in terms of changes in body weight in response to exercise training. The median (range) total sample size of the included studies was 53 (3–439). The median (range) age was 37 (20–62) years. Forty‐six studies reported BMI at baseline, with the median (range) being 30.6 (27.0–38.4) kg/m^2^. Males and females were included in 26 studies,^6^, ^7^, ^16^, ^29^, ^30^, ^31^, ^34^, ^35^, ^36^, ^37^, ^38^, ^39^, ^40^, ^41^, ^42^, ^43^, ^44^, ^45^, ^46^, ^47^, ^48^, ^49^, ^50^, ^51^, ^52^, ^53^ males only in eight studies,^27^, ^28^, ^32^, ^54^, ^55^, ^56^, ^57^, ^58^ and females only in 14 studies^25^, ^59^, ^60^, ^61^, ^62^, ^63^, ^64^, ^65^, ^66^, ^67^, ^68^, ^69^, ^70^, ^71^; the overall median percentage of females was 67% (0%–100%). Five studies included subjects with comorbidities: three with prediabetes,^42^, ^43^, ^44^ one with metabolic syndrome,^57^ and one with dyslipidemia.^34^

Interventions are described in detail in Table 1. Duration of exercise training ranged from 2 to 72 weeks, with a median duration of 12 weeks, on median 5 (2–7) days/week. Endurance/aerobic training was performed in 43 studies,^6^, ^7^, ^16^, ^25^, ^27^, ^28^, ^29^, ^30^, ^34^, ^35^, ^36^, ^37^, ^38^, ^39^, ^40^, ^41^, ^44^, ^45^, ^46^, ^47^, ^48^, ^49^, ^50^, ^51^, ^53^, ^54^, ^55^, ^56^, ^57^, ^58^, ^59^, ^60^, ^61^, ^62^, ^63^, ^64^, ^65^, ^66^, ^67^, ^68^, ^69^, ^70^, ^71^ resistance training in five studies,^34^, ^42^, ^43^, ^52^, ^55^ a combination of aerobic and resistance training in one study,^34^ and high‐intensity interval training/intermittent exercise in five studies.^31^, ^32^, ^44^, ^48^, ^58^ Exercise duration was prescribed in minutes or energy expenditure (kcal), or relative to maximal aerobic capacity (VO_2max_) or heart rate (%HR_max_, %HR_reserve_, or ventilatory threshold [HR_VT_]). The median (range) exercise prescription was 45 (30–111) min or 500 (233–600) kcal per session at 70% (40%–75%) VO_2max_, 70% (60%–78%) HR_max_, or 60% (55%–75%) HR_reserve_. Exercise sessions were fully supervised in 32 studies, partially supervised in six studies, not supervised in eight studies, and not reported in two studies.

Settings included free‐living (21 studies), laboratory (17 studies), or a combination of the two (10 studies). Energy intake was reported in 43 studies (as daily energy intake [38 studies; 31 included in meta‐analysis], single test meal intake [four studies; three included in meta‐analysis], preload‐test meal paradigm [two studies]), and appetite ratings in 19 studies (10 and nine included in meta‐analysis on fasting hunger and fullness, respectively). Three studies used the satiety quotient to assess the strength of satiety, which is calculated by dividing the change in hunger ratings before and after the test meal by the amount of consumed energy at the test meal (mm/kcal).^72^ Eating behavior traits were reported in nine studies (eight, eight, and six included in meta‐analysis on restraint, disinhibition/uncontrolled eating, and susceptibility to hunger measured by the Three‐Factor Eating Questionnaire/Eating Inventory,^73^, ^74^ respectively) and food reward in seven studies. In the 31 studies assessing daily energy intake included in the meta‐analysis, four measured it in the laboratory objectively, 22 used self‐reported measures, four used a combination of objective and self‐reported measures and one study calculated it from doubly labeled water.

Except for one study which had a 6‐month no‐contact follow‐up,^43^ all studies assessed outcomes immediately after the intervention.

### 3.2 Study quality

Overall, study quality was rated as poor, fair, and good in 39 (81%), seven (15%), and two (4%) studies, respectively (Table S2). The main quality issues pertained to not properly reporting randomization or blinding methods, drop‐out rate >20% or did not report it, not using valid and reliable assessment of outcome measures (e.g., for energy intake), not performing ITT analyses, or not having a sample size justification. Forty of the 48 studies reported a high level of adherence.

### 3.3 Study findings

The findings of the included studies are presented in Table S3.

#### 3.3.1 Energy intake

##### Exercise versus control groups

**FIGURE 2:**
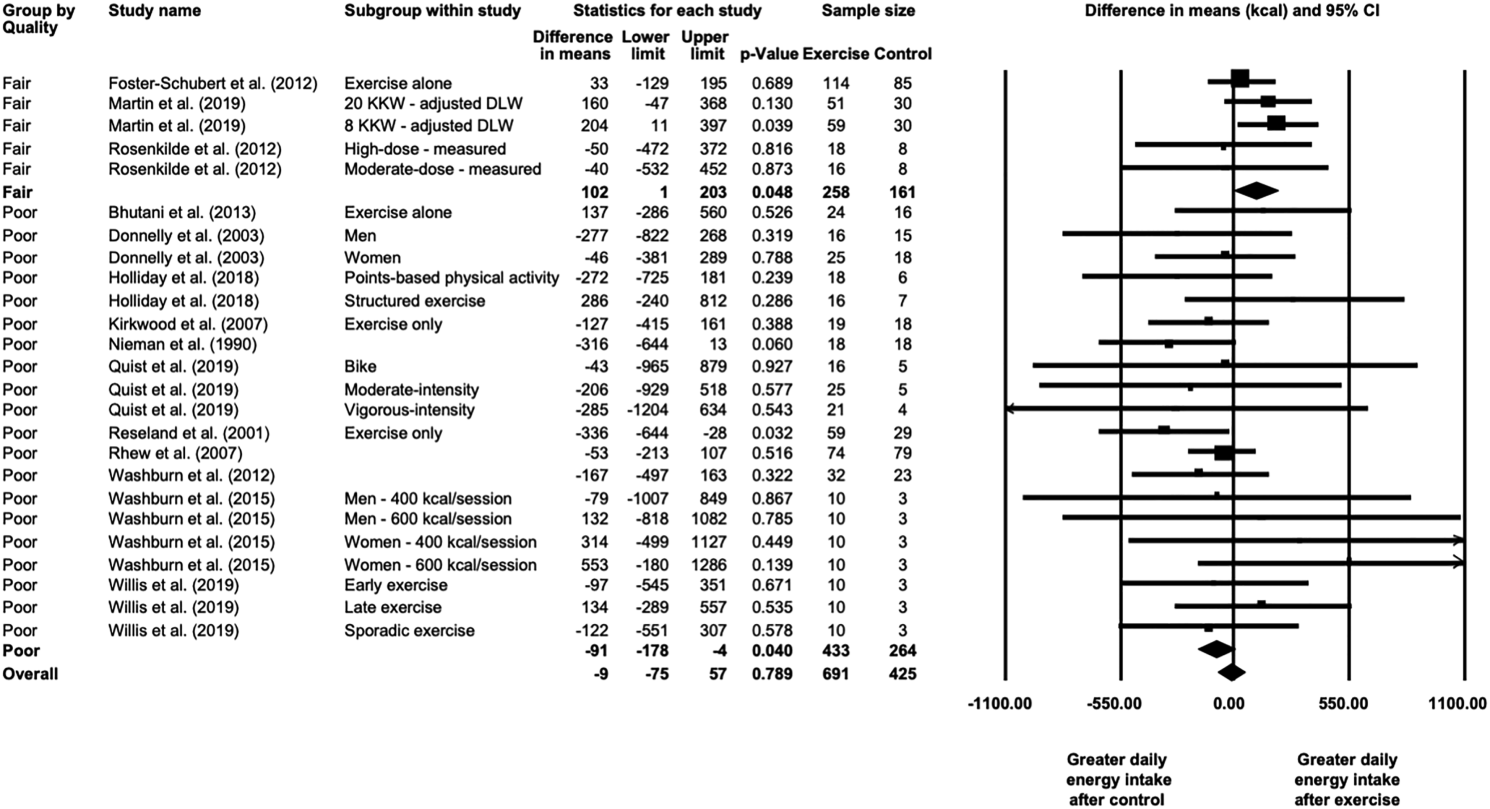
Forest plot of differences in postintervention daily energy intake between exercise and no‐exercise control groups, grouped by study quality (*N* = 25 study arms)

In 14 studies reporting daily energy intake that included a nonexercising control group, a meta‐analysis was performed to compare postintervention daily energy intake between exercise and control groups. Meta‐analysis of 25 study arms (exercise *N* = 691 and control *N* = 425) showed no postintervention difference between exercise and control groups (MD = −13 [−83, 58] kcal; *p* = 0.721). Heterogeneity was low (*I* ^2^ = 6%, *Q* = 25, *p* = 0.383). Sensitivity analysis with the one‐study‐removed procedure did not show any impact of a single study on the overall effect. The difference increased when only fair/good quality studies (three of 14 studies; Figure 2) were included in the analysis (*N* = 5 study arms, exercise *N* = 258, control *N* = 161; MD = 102 [1, 203] kcal, *p* = 0.048), and heterogeneity was very low (*I* ^2^ = 0%, *Q* = 3, *p* = 0.576). However, the effect size was negligible (SMD = 0.178 [−0.020, 0.377]). Visual inspection of the funnel plot (Figure S1) suggested little evidence of publication bias. The trim‐and‐fill method suggested four missing studies to the right (adjusted MD = 23 [−58, 104] kcal) but no presence of publication bias with Egger's regression (*p* = 0.492).

##### Exercise groups only

**FIGURE 3:**
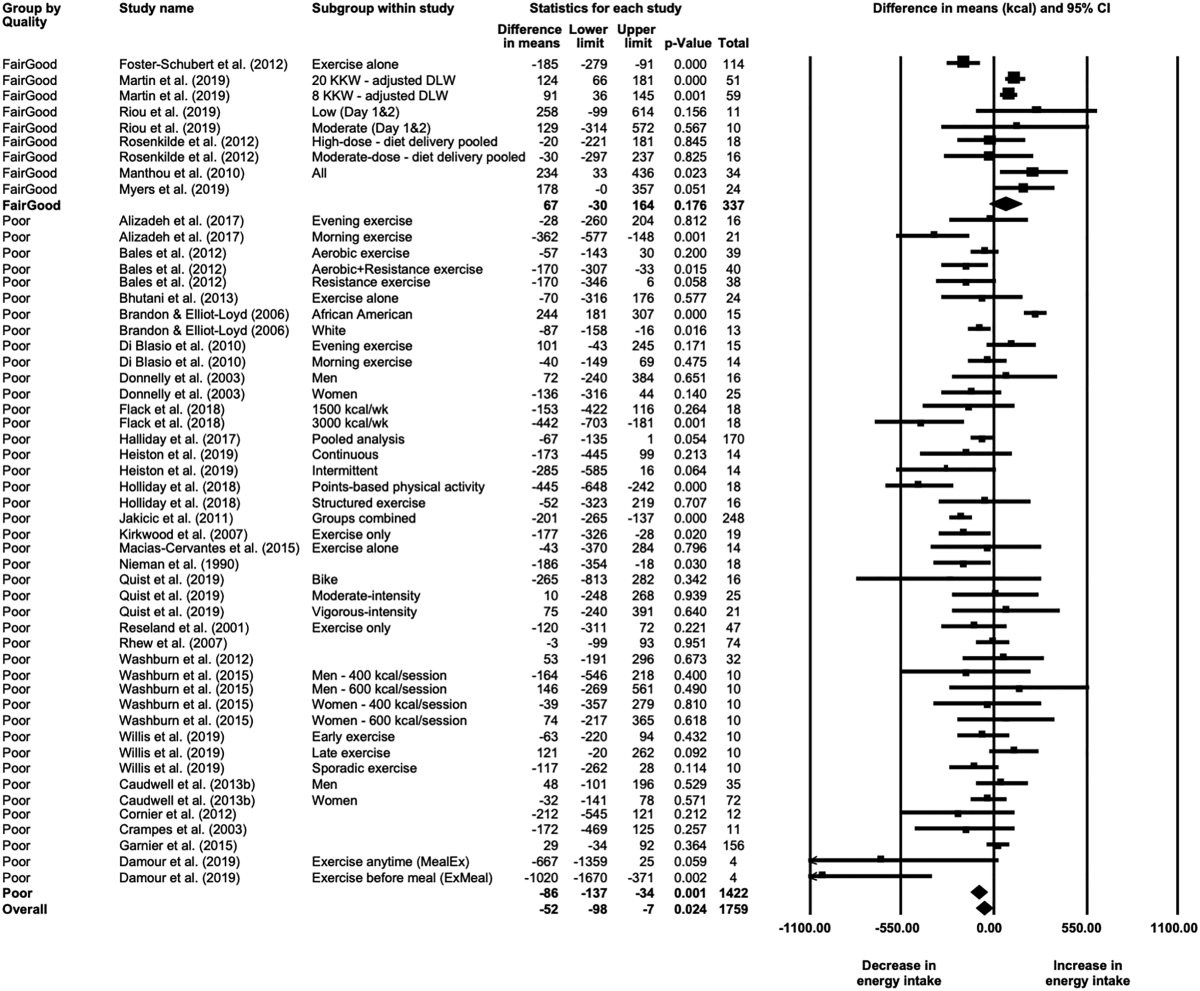
Forest plot of changes in daily energy intake from baseline to after exercise training in individuals with overweight or obesity, grouped by study quality (*N* = 52 study arms)

Meta‐analysis of 31 studies (52 study arms) demonstrated a significant decrease in mean daily energy intake after exercise training (*N* = 1759; MD = −57 [−104, −11] kcal, *p* = 0.016). The effect size was negligible (SMD = −0.09 [−0.17, −0.004]). Heterogeneity among studies was high (*I* ^2^ = 80%, *Q* = 259, *p* < 0.001). Sensitivity analysis with the one‐study‐removed procedure did not show any impact of a single study on the overall effect. Considering the large number of poorly rated studies, the analysis was also run in only the six fair/good quality studies (nine study arms; Figure 3) and showed that the effect was nullified (*N* = 337; MD = 67 [−30, 164] kcal, *p* = 0.176), with a negligible effect size (SMD = 0.185 [−0.067, 0.437]) and high heterogeneity (*I* ^2^ = 79%, *Q* = 39, *p* < 0.001). As shown in Table S4, there was no effect of sex or exercise dose/intensity. There was an effect of energy intake method, with self‐reported energy intake and doubly labeled water (i.e., calculated energy intake) reporting opposite effects (i.e., reduction of 111 kcal vs. increase of 106 kcal, respectively, although the latter only included two arms of the same study). Meta‐regression showed no relationship between intervention duration and changes in daily energy intake (*β* = −0.567 [−3.039, 1.905], *p* = 0.653) and was not impacted when the data from Damour et al.,^31^ with differences in means of −667 and −1020 kcal, were removed (*β* = −1.053; *p* = 0.392). Visual inspection of the funnel plot (Figure S2) suggested some publication bias, with the trim‐and‐fill method suggesting two missing studies to the right (adjusted MD = −51 [−98, 4] kcal) and a significant Egger's regression (intercept = −1.247 [−2.308, −0.187], *p* = 0.022).

A separate meta‐analysis was performed for daily and test meal energy intake combined (58 study arms), showing a negligible effect toward a reduction in energy intake after exercise training (*N* = 1770, SMD = −0.092 [−0.171, −0.013], *p* = 0.022). Heterogeneity among studies was moderate (*I* ^2^ = 64%, *Q* = 159, *p* < 0.001). Sensitivity analysis with the one‐study‐removed procedure did not show any impact of a single study on the overall effect. As shown in Figure S3, when only the fair/good studies were considered (13 study arms), the effect was nullified (*N* = 338; SMD = 0.114 [−0.082, 0.310], *p* = 0.253). Moderator analyses (Table S5) revealed no effect of sex, exercise dose/intensity, nor energy intake type (daily vs. single test meal). An effect of energy intake method was observed, with self‐reported energy intake and doubly labeled water (i.e., calculated energy intake) reporting opposite effects (i.e., reduction vs. increase, respectively), although the latter only included two arms of the same study. Visual inspection of the funnel plot (Figure S4) suggested unlikely presence of publication bias, with the trim‐and‐fill method suggesting one missing study to the right (adjusted SMD = −0.087 [−0.166, 0.008]) and Egger's test suggesting no evidence of publication bias (*p* = 0.256).

#### 3.3.2 Appetite ratings

**FIGURE 4:**
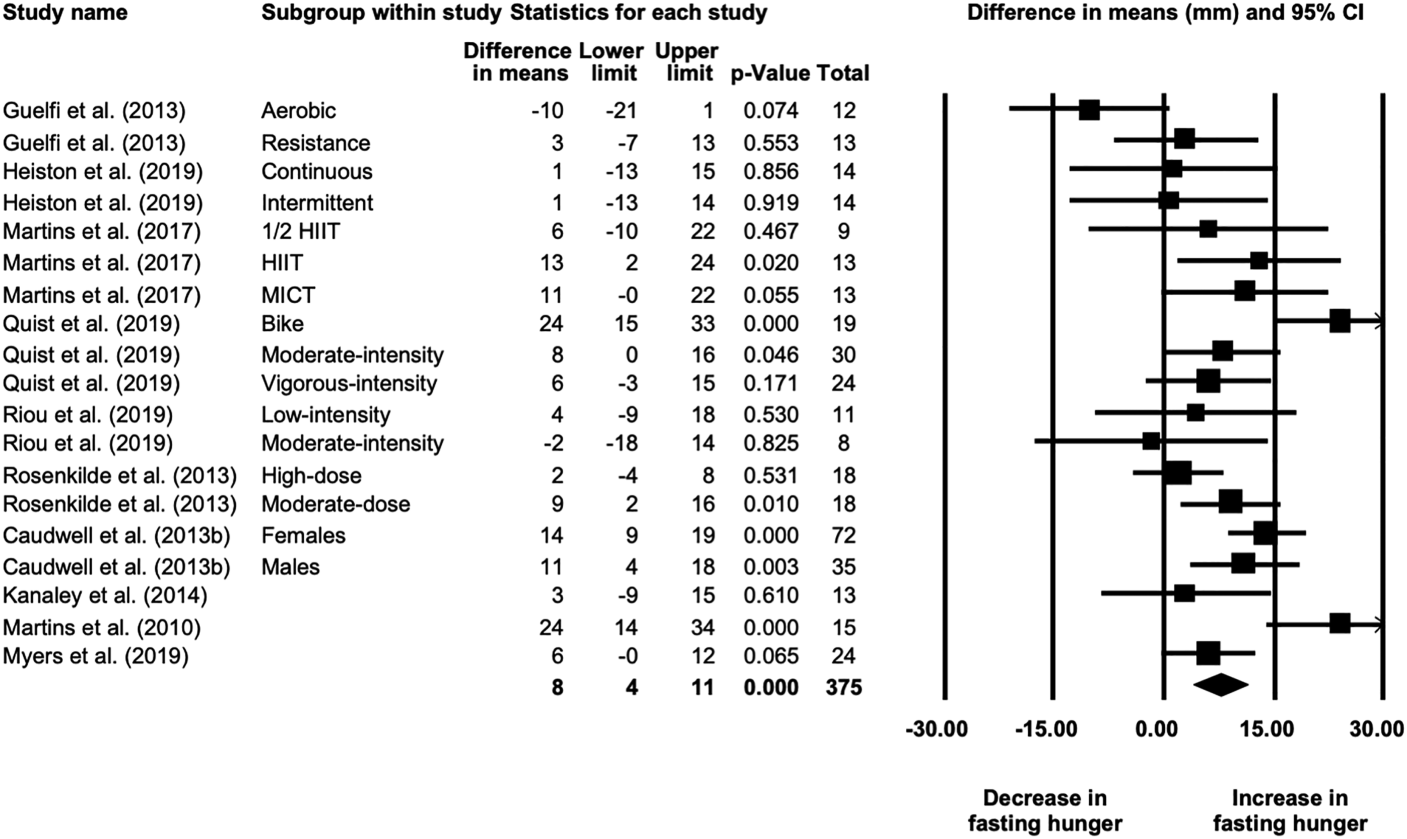
Forest plot of changes in fasting hunger showing an overall small increase after exercise training in individuals with overweight or obesity (*N* = 19 study arms)

Meta‐analysis of 10 studies showed that fasting hunger increased by 8 (4, 11) mm in response to exercise training (19 study arms, *N* = 375; *p* < 0.001; Figure 4). The effect size was small (SMD = 0.327 [0.183, 0.471]). Heterogeneity was moderate (*I* ^2^ = 64%, *Q* = 49, *p* < 0.001). The effect did not differ by sex (*p* = 0.409) and was not influenced by intervention duration (range 2–24 weeks; *β* = 0.482 [−0.101, 1.065], *p* = 0.105). The one‐study‐removed procedure did not show any impact of a single study on the overall effect. The effect persisted in studies rated as fair/good (three studies, five study arms, *N* = 79; MD = 5 [2, 9] mm, *p* = 0.005). The funnel plot (Figure S5) suggested potential evidence of publication bias, with the trim‐and‐fill method suggesting four missing studies to the right (adjusted MD = 10 [6, 13] mm), but Egger's test was nonsignificant (p = 0.283). Of the 13 studies reporting changes in postprandial or daily hunger, eight found no changes and four found increases (see Table S3).

Meta‐analysis of nine studies showed that fasting fullness did not change in response to exercise training (17 study arms, *N* = 268; MD = 1 [−3, 5] mm, *p* = 0.641; Figure S6). Heterogeneity was high (*I* ^2^ = 80%, *Q* = 82, *p* < 0.001). The effect did not differ by sex (*p* = 0.219) and was not influenced by intervention duration (range 2 to 24 weeks; *β* = −0.137 [−0.763, 0.489], *p* = 0.668). The one‐study‐removed procedure did not show any impact of a single study on the overall effect. The lack of effect persisted in studies rated as fair/good (three studies, five study arms, *N* = 79; MD = 2 [−8, 11] mm; *p* = 0.736). The funnel plot (Figure S7) suggested little evidence of publication bias; however, the trim‐and‐fill method suggested three missing studies to the right (adjusted MD = 4 [−1, 8] mm), but Egger's test was nonsignificant (*p* = 0.797). Of the 11 studies reporting postprandial or daily fullness, seven found no changes whereas two reported increases or decreases (see Table S3).

#### 3.3.3 Eating behavior traits and food reward

**FIGURE 5:**
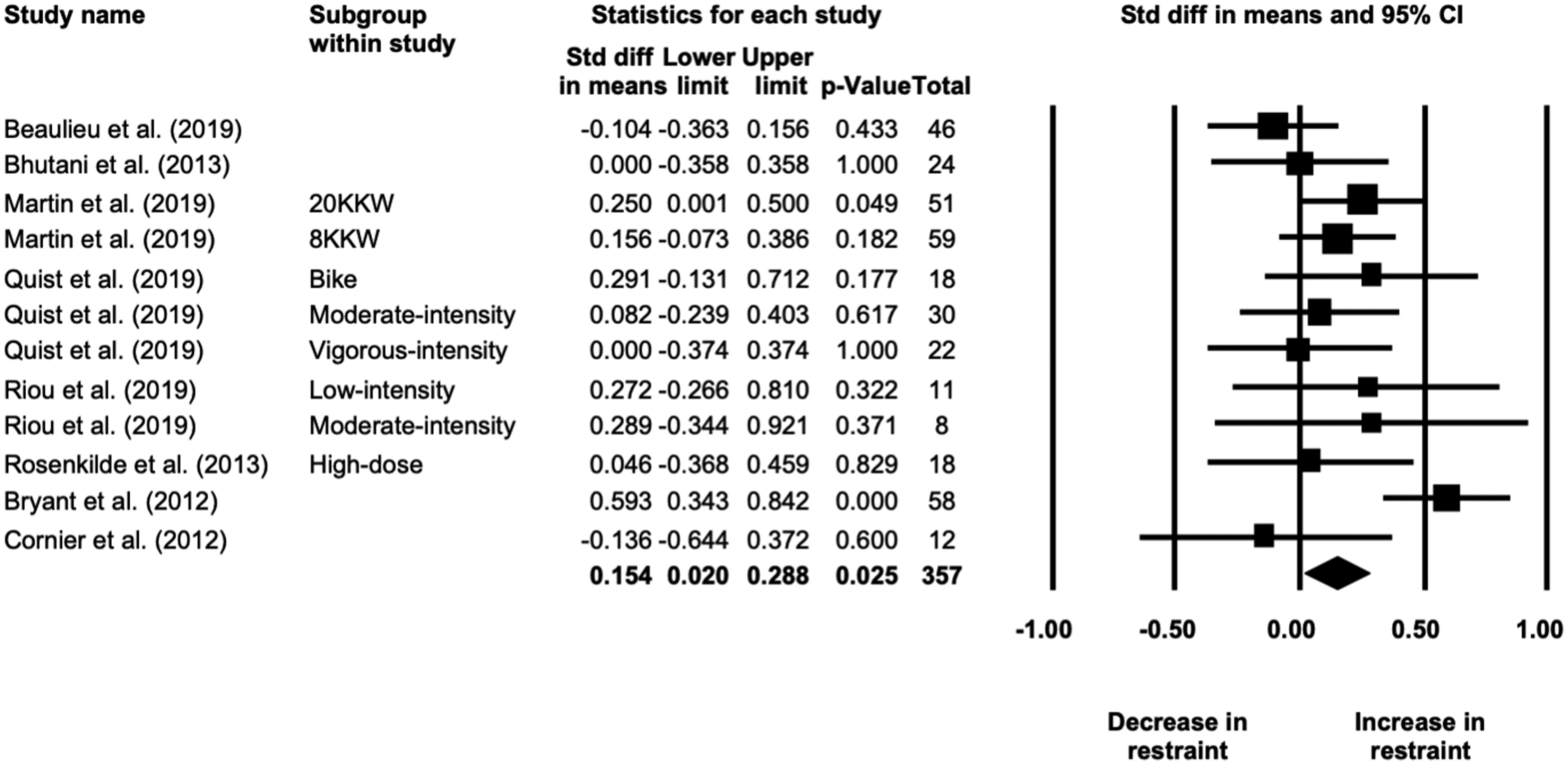
Forest plot of changes in dietary restraint showing an overall significant but negligible increase after exercise training in individuals with overweight or obesity (*N* = 12 study arms)

Meta‐analysis of eight studies showed that restraint did not change in response to exercise training (13 study arms, *N* = 375; SMD = 0.074 [−0.109, 0.256], *p* = 0.430). Heterogeneity was moderate (*I* ^2^ = 71%, *Q* = 42, *p* < 0.001). The one‐study‐removed procedure showed that the overall effect was influenced by one outlier (moderate‐dose arm of Rosenkilde et al.^28^). Without that study arm included, there was a significant but negligible increase in restraint (*N* = 357; SMD = 0.154 [0.020, 0.288], *p* = 0.025; Figure 5). Heterogeneity was moderate (*I* ^2^ = 45%, *Q* = 20, *p* = 0.046). The effect strengthened slightly in studies rated as fair/good (three studies, five study arms, *N* = 147; SMD = 0.190 [0.044, 0.336], *p* = 0.011). The funnel plot (Figure S8) suggested little evidence of publication bias, with the trim‐and‐fill method suggesting two missing studies to the right (adjusted SMD = 0.206 [0.070, 0.341]), but Egger's test was nonsignificant (*p* = 0.533).

**FIGURE 6:**
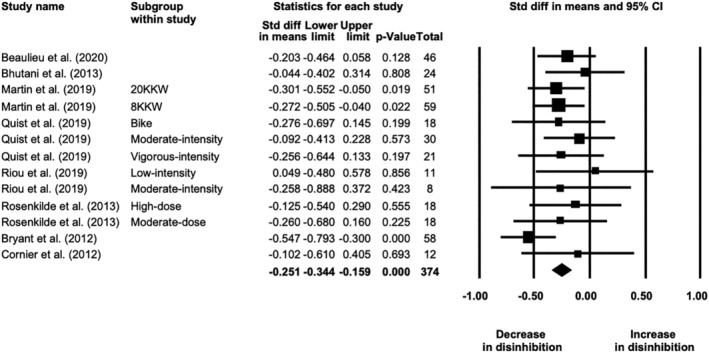
Forest plot of changes in disinhibition/uncontrolled eating showing an overall small decrease after an exercise intervention in individuals with overweight or obesity (*N* = 13 study arms)

Meta‐analysis of eight studies showed a small but significant decrease in disinhibition/uncontrolled eating in response to exercise training (13 study arms, *N* = 374; SMD = −0.251 [−0.344, −0.159], *p* < 0.001; Figure 6). Heterogeneity was very low (*I* ^2^ = 0%, *Q* = 10, *p* = 0.617). The one‐study‐removed procedure did not show any impact of a single study on the overall effect. The effect persisted in studies rated as fair/good (three studies, six study arms, *N* = 165; SMD = −0.240 [−0.379, −0.102], *p* = 0.001). The funnel plot (Figure S9) suggested some presence of publication bias, with the trim‐and‐fill method suggesting six missing studies to the left (adjusted SMD = −0.337 [−0.429, −0.245]) and Egger's test approaching significance (*p* = 0.084).

Meta‐analysis of six studies showed no changes in susceptibility to hunger in response to exercise training (11 study arms, *N* = 339; SMD = −0.014 [−0.142, 0.114], *p* = 0.831; Figure S10). Heterogeneity was moderate (*I* ^2^ = 38%, *Q* = 16, *p* = 0.100). The one‐study‐removed procedure did not show any impact of a single study on the overall effect. The lack of effect persisted in studies rated as fair/good (three studies, six study arms, *N* = 165; SMD = 0.062 [−0.158, 0.282], *p* = 0.580). The funnel plot (Figure S11) suggested no publication bias, with the trim‐and‐fill method suggesting no missing studies and Egger's test being nonsignificant (*p* = 0.449).

Regarding food reward, three of four studies reporting food liking found no changes in response to the exercise interventions,^35^, ^48^, ^69^ whereas Martin et al.^7^ found a decrease in preference for high‐fat/high‐carbohydrate foods in the high‐dose exercise group compared with the moderate‐dose exercise group. In five studies reporting either implicit wanting (Leeds Food Preference Questionnaire), neuronal activation to food cues (fMRI), or relative food reinforcement, four found a decrease in response to exercise interventions,^35^, ^41^, ^53^, ^69^ although the effect was nullified when controlling for desire to eat in one of the studies,^41^ whereas another found no changes.^48^ In three studies assessing the food reward response to acute exercise before and after training, one found decrease in liking for savory food after aerobic exercise,^69^ another found a tendency for an increase in liking for high‐fat savory foods after MIIT but a decrease after HIIT,^32^ whereas one found no changes in the neuronal response to food cues with acute exercise postintervention.^53^ For more details, see Table S3.

## 4 DISCUSSION

The outcomes of this systematic review with meta‐analysis suggest that the imposition of an exercise training regime in people with overweight or obesity does not—on the average—induce any substantial change in food intake or appetite during the period of training. Contrary to what is often believed—namely, that performing exercise will drive up energy intake to nullify the increase in energy expenditure—the meta‐analyses limited to fair/good quality studies actually showed no significant change in energy intake pre‐post training (67 kcal) and a small (102 kcal) but negligible (in terms of effect size) postintervention difference compared with no‐exercise control groups. It is important to note that these findings need to be interpreted within the limitations imposed by self‐reported food intake measures and the limited number of studies rated as fair or good quality (~20%). Some differences in effects (increases vs. decreases) were noted in the subgroup analyses by daily energy intake method (Table S4); however, these, as well as the overall effects observed, fall within the precision limits of these assessment methods (e.g., coefficient of variation of 5% for doubly labeled water^75^ and 23% for self‐reported energy intake^76^) and thus need to be interpreted cautiously. Changes in energy intake were not influenced by the sex of the participants or the dose/intensity of the exercise intervention. The lack of a major impact of exercise training on average energy intake observed is in line the systematic review not specific to individuals with overweight or obesity by Donnelly et al.^21^ Furthermore, effects small in magnitude were observed for an increase in fasting hunger and a decrease in disinhibition, as well as a negligible increase in dietary restraint. Because these data demonstrate that on average, a planned and deliberate exercise intervention does not stimulate appetite to any meaningful degree nor compromise energy balance, it would be expected that an exercise intervention should lead to some degree of weight and fat loss. This was demonstrated in our sister overview of systematic reviews and meta‐analyses on changes in body composition by Bellicha et al.,^77^ which observed an overall reduction in body weight ranging from −0.8 to −3.5 kg and fat mass ranging from −1.3 to −2.6 kg after aerobic exercise training in individuals with overweight or obesity.

It should be considered that the absence of any noticeable pre‐post effect of exercise training on energy intake in the majority of studies or to the negligible increase compared with controls in the higher rated studies could be due to a number of factors—mechanistic and methodological. First, any effect on the mechanisms of appetite control would invoke the dual action on appetite control described earlier by King et al.^47^ This dual action comprises an increase in early day hunger but accompanied by an increase in the strength of episodic satiety signaling. A simultaneous and equal effect on these two processes would leave net energy intake unaltered. Indeed, this model of appetite control is supported by the current review with the small increase in fasting hunger observed. Furthermore, in the small number of studies assessing energy compensation in response to a preload—a paradigm to assess the strength of satiety—an improvement was shown after exercise training,^50^, ^58^ as well as in studies assessing the satiety response to food via the satiety quotient.^38^, ^47^

Another mechanism by which exercise could affect eating behavior is by exerting “spill‐over effects” to influence food choices and food intake, as also suggested by some studies included in this review.^35^, ^41^, ^43^, ^45^, ^64^ This is supported by the synthesis of the eating behavior traits, which found a small decrease in dietary disinhibition/uncontrolled eating after exercise training, as well as an increase, albeit of negligible magnitude, in dietary restraint. A small number of studies in the current review also suggest that exercise could also improve food reward/preferences^7^, ^35^, ^41^, ^53^, ^69^; this has recently been reviewed extensively elsewhere by Beaulieu et al.^78^ Therefore, exercise training could lead to a reduction in the susceptibility to overconsumption.

Interestingly, in the included studies that had energy‐reduced diet or combined diet and exercise groups, any changes in energy intake with exercise‐only interventions were minimal compared with the dietary interventions. The current literature suggests that performing exercise when diet is free to vary has relatively small effects on overall eating behavior in individuals with overweight or obesity. However, as stated above and in prior work,^19^, ^79^ it appears that regular exercise enhances the sensitivity of the appetite control system. Exercise could also reduce compensatory effects seen with dietary energy restriction alone.^80^, ^81^, ^82^ It is likely that dietary energy restriction would be necessary alongside exercise training for a maximal impact on energy intake and eating behaviors, but this is beyond the scope of the current review. Moreover, dietary recommendations are likely to vary depending on individual goals—weight loss, weight maintenance, management of comorbidities, and so forth. Further research is required to find the optimal combination of exercise and dietary prescriptions for obesity management. In addition, we want to note that energy flux is an important variable, with a high energy flux generating better control of food behavior (e.g., Hägele et al.^83^).

A number of methodological comments are in order. First, for the measures of food intake, most studies relied on diary recordings or some form of self‐report. There is ample evidence^84^, ^85^ that such measures are prone to misreporting and cannot be regarded as truly representative of actual food consumed.^86^ This is particularly important when the differences between two conditions (before vs. after; exercise vs. no‐exercise control, for example) are likely to be small. This, however, is unlikely to be the reason for the failure to detect any effect of exercise training since when the analysis was repeated using only the ‘good’ and ‘fair’ quality studies, negligible effects were observed. Indeed, we rechecked the included papers in the energy intake meta‐analysis for those that included changes in body composition and objectively measured energy expenditure and identified five studies (10 study arms)^7^, ^30^, ^41^, ^67^, ^69^ in which to calculate changes in energy intake using an energy balance equation.^9^ The median (range) pre‐post change in calculated daily energy intake obtained was 70 kcal (−381 to 174). While the range was quite large, the median was surprisingly similar to the overall result of the pre‐post change in energy intake from the meta‐analysis of fair/good studies (nine study arms) of 67 kcal (95% CI −30, 164). Thus, this supplementary analysis supports our main findings. Second, there was also a very large range in duration of the interventions included (from 2 to 72 weeks); however, meta‐regression found no influence of the intervention duration on the effects observed. Third, there were not enough studies/subgroups included to determine whether exercise mode (aerobic, resistance, HIIT) differently affected food intake, as most studies included used aerobic exercise protocols. Thus, more studies are required to examine the influence of different exercise training modalities on energy intake and appetite control. Other parameters of interest that were included in the current studies but require more research include exercise dose and intensity, exercise timing (morning vs. evening^30^, ^59^, ^61^ or in relation to meals^31^), and compensation status with regards to predicted and actual weight loss (compensators/nonresponders vs. noncompensators/responders).^6^, ^7^, ^37^, ^47^, ^66^ Fourth, at the time of peer‐review, the search had been over a year old and three new studies were identified.^87^, ^88^, ^89^ The findings are briefly reported here.

In their secondary analysis of a 12‐week aerobic exercise intervention of either six sessions per week, two sessions per week, or no‐exercise control, Flack et al. found no significant changes in the reinforcing value of healthy and unhealthy snack foods (i.e., food reward).^87^ Mason et al. found no changes in eating behavior traits relating to binge eating, uncontrolled eating, emotional eating, and restrained eating after 12 months of aerobic exercise training in postmenopausal women.^88^ And Paravidino et al. showed that during 2 weeks of moderate‐intensity, vigorous‐intensity, or no‐exercise control in Brazilian Naval Academy cadets with overweight, changes in self‐reported energy intake were not different among groups nor were there any changes in appetite sensations taken before and after an ad libitum cafeteria breakfast.^89^

A final issue concerns the methodology of meta‐analyses. Although in these statistical processes the effects of studies on mean outcomes are standardized to account for the variance in the range of individual scores, the fundamental measure remains the *average* of the group of participants. As certain statisticians have pointed out, “the average is an abstraction, reality is variation.”^90^ The average is only one measure of the outcome of a period of exercise training. Accordingly, it has invariably been observed that, following an imposed period of exercise, individual variability is very large.^6^, ^7^, ^8^ Participants may react in quite different ways to the physiological and psychological demands of exercise. Consequently, although our analyses show no change in the “average of averages” (meta‐analysis), this cannot be regarded as a prediction of the likely outcome for every individual. People will still show widely divergent responses to exercise even though the mean does not change. The interpretation of the outcomes of systematic reviews and meta‐analyses has to be made with prudence. However, what can be deduced from the current review is that there is no pronounced overall effect of exercise interventions on energy intake or appetite, but some negligible‐to‐small effects were observed. In turn, this is positive news for anticipating a beneficial effect of exercise training on negative energy balance and fat loss.

Finally, the effect of a deliberately imposed exercise regime in inactive individuals with overweight or obesity should be considered in the context of an energy balance framework for appetite control. A growing body of evidence indicates that energy expenditure can be regarded as a driver of energy intake (e.g., Blundell et al.^91^ and Lam and Ravussin^92^). However, physical activity energy expenditure, as a lifestyle component of total daily energy expenditure, reflects a higher level of bodily activity and energy expenditure distributed across the day through a variety of behaviors. This situation exerts a mild tonic effect on appetite and is usually associated with leanness.^79^ This can be contrasted with the introduction of daily sessions of exercise in a sedentary/inactive person with obesity, which represents a severe jolt to physiology; this was the focus of the present analyses. To achieve weight loss (i.e., negative energy balance), energy expenditure needs to be greater than energy intake; therefore, any small increase in energy intake would be required to be less than the prescribed exercise energy expenditure. Furthermore, while this review focused solely on the intake side of energy balance, it is important to consider that exercise training may affect other components of energy expenditure such as RMR and nonexercise physical activity to influence energy balance.^5^, ^9^

The outcome of this review has demonstrated that, subject to the reservations noted above, people with overweight or obesity may undertake exercise training without fear that there will be an inevitable large increase in appetite and energy intake and as shown in our sister review,^77^ with the expectation that the exercise sessions will result in a negative energy balance which, in turn, will lead to some loss of adipose tissue.

## CONFLICT OF INTEREST

No conflict of interest statement.

## AUTHOR CONTRIBUTIONS

KB and JB performed the literature search, study selection, data extraction, and quality assessment. KB performed the meta‐analysis. All authors participated in the interpretation of data. KB and JB drafted the manuscript, and authors critically revised the manuscript.

## Supporting information

**Table S1.** Keywords included in database search strategy
**Table S2**. Summary of quality assessment of included studies
**Table S3**. Findings of included studies
**Table S4.** Moderator and subgroup analyses pre‐post changes in daily energy intake in exercise groups only
**Table S5.** Moderator and subgroup analyses for pre‐post changes in daily and test meal energy intake combined in exercise groups only
**Figure S1.** Funnel plot of post‐intervention comparisons in daily energy intake between exerciser and no‐exercise control groups (N = 25 study arms).
**Figure S2.** Funnel plot of pre‐post changes in daily energy intake (N = 52 study arms).
**Figure S3.** Forest plot of pre‐post changes in daily and test meal energy intake in individuals with overweight or obesity, grouped by study quality (N = 58 study arms).
**Figure S4.** Funnel plot of pre‐post changes in daily and test meal energy intake (N = 58 study arms).
**Figure S5.** Funnel plot of pre‐post changes in fasting hunger (N = 19 study arms)
**Figure S6.** Forest plot of changes in fasting fullness showing no overall change after exercise training in individuals with overweight or obesity (N = 17 study arms).
**Figure S7.** Funnel plot of pre‐post changes in fasting fullness (N = 17 study arms)
**Figure S8.** Funnel plot of pre‐post changes in dietary restraint (N = 12 study arms)
**Figure S9.** Funnel plot of pre‐post changes in disinhibition/uncontrolled eating (N = 13 study arms)
**Figure S10.** Forest plot of changes in susceptibility to hunger showing no overall change after exercise training in individuals with overweight or obesity (N = 11 study arms).
**Figure S11.** Funnel plot of pre‐post changes in susceptibility to hunger (N = 11 study arms)
