# Supplementary material for: Effect of exercise training interventions on energy intake and appetite control in adults with overweight or obesity: A systematic review and meta‐analysis
Source: Obes Rev. 2021 May 5;22(Suppl 4):e13251. doi: 10.1111/obr.13251 (PMC8365695; doi:10.1111/obr.13251)
Supplement: Supplementary file 1 — Table S1. Keywords included in database search strategy Table S2. Summary of quality assessment of included studies Table S3. Findings of included studies Table S4. Moderator and subgroup analyses pre‐post changes in daily energy intake in exercise groups only Table S5. Moderator and subgroup analyses for pre‐post changes in daily and test meal energy intake combined in exercise groups only Figure S1. Funnel plot of post‐intervention comparisons in daily energy intake between exerciser and no‐exercise control groups (N = 25 study arms). Figure S2. Funnel plot of pre‐post changes in daily energy intake (N = 52 study arms). Figure S3. Forest plot of pre‐post changes in daily and test meal energy intake in individuals with overweight or obesity, grouped by study quality (N = 58 study arms). Figure S4. Funnel plot of pre‐post changes in daily and test meal energy intake (N = 58 study arms). Figure S5. Funnel plot of pre‐post changes in fasting hunger (N = 19 study arms) Figure S6. Forest plot of changes in fasting fullness showing no overall change after exercise training in individuals with overweight or obesity (N = 17 study arms). Figure S7. Funnel plot of pre‐post changes in fasting fullness (N = 17 study arms) Figure S8. Funnel plot of pre‐post changes in dietary restraint (N = 12 study arms) Figure S9. Funnel plot of pre‐post changes in disinhibition/uncontrolled eating (N = 13 study arms) Figure S10. Forest plot of changes in susceptibility to hunger showing no overall change after exercise training in individuals with overweight or obesity (N = 11 study arms). Figure S11. Funnel plot of pre‐post changes in susceptibility to hunger (N = 11 study arms) [file OBR-22-e13251-s001.pdf]

Supporting Information - Beaulieu et al. Effect of Exercise Training Interventions on Energy Intake and Appetite Control in Adults with Overweight or Obesity: A Systematic Review and Meta-Analysis
k.beaulieu@leeds.ac.uk

Table S1. Keywords included in database search strategy

**Obesity**	**Physical activity**	**Age**	**Energy intake**	**Appetite control**
Overweight<br>Obesity<br>Obese	Physical activit*<br>Exercise<br>Sport<br>Endurance activity<br>Endurance activities<br>Aerobic activity<br>Aerobic activities<br>Cardiovascular activit*<br>Resistance training<br>Strength training<br>Muscle-strengthening<br>Weight-Lifting program<br>High-intensity interval training<br>HIIT<br>Physical conditioning<br>Walking<br>Sedentary time<br>Sedentary lifestyle<br>Sitting time	Adults<br>(NOT child, children, adolescents, pediatric)	Energy intake/<br>Diet/<br>Calori* intake<br>Food intake<br>Meal size<br>Energy compensation<br>Energy density<br>Dietary protein/<br>Dietary fat/<br>Dietary carbohydrate/<br>Macronutrient	Appetite/<br>Feeding behavio*/<br>Food preference*/<br>Eating behavio*<br>Hunger<br>Satiety<br>Satiation<br>Fullness<br>Motivation to eat<br>Food choice<br>Food selection<br>Desire to eat<br>Palatability<br>Food reward<br>Hedonic<br>Liking<br>Wanting<br>Food craving*

Supporting Information - Beaulieu et al. Effect of Exercise Training Interventions on Energy Intake and Appetite Control in Adults with Overweight or Obesity: A Systematic Review and Meta-Analysis
k.beaulieu@leeds.ac.uk

Table S2. Summary of quality assessment of included studies

References	Criteria														Total "Yes"	Total "No"	Total "other"	Quality rating
	1	2	3	4	5	6	7	8	9	10	11	12	13	14				
Randomized or controlled trials																		
Alizadeh et al. (2017)	Yes	Yes	NR	Yes	Yes	Yes	No	Yes	NR	NA	No	Yes	Yes	No	8	3	3	Poor
Bales et al. (2012)	Yes	Yes	NR	NR	NR	Yes	No	NR	Yes	NA	No	No	Yes	No	5	4	5	Poor
Beaulieu et al. (2020)	No	NA	NA	No	No	Yes	NR	NR	Yes	NA	Yes	Yes	Yes	Yes	6	3	5	Poor
Bhutani et al. (2013)	Yes	NR	NR	NR	NR	Yes	No	No	Yes	NA	No	No	Yes	Yes	5	4	4	Poor
Brandon & Elliot-Loyd (2006)	Yes	NR	NR	NR	NR	No	No	No	Yes	NA	No	Yes	Yes	No	4	5	4	Poor
Di Blasio et al. (2010)	No	No	No	No	NR	Yes	No	NR	Yes	NA	No	No	CD	No	2	8	4	Poor
Donnelly et al. (2003)	Yes	Yes	No	No	NR	Yes	No	NR	Yes	NA	Yes	No	Yes	No	6	5	3	Poor
Dorling et al. (2019)	Yes	NR	NR	NR	NR	No	Yes	Yes	NR	NA	Yes	No	Yes	No	5	3	6	Fair
Flack et al. (2018)	Yes	Yes	Yes	No	No	Yes	Yes	NR	Yes	NA	No	No	Yes	No	7	5	2	Poor
Foster-Schubert et al. (2012)	Yes	Yes	NR	NR	Yes	Yes	Yes	Yes	Yes	NA	No	Yes	Yes	Yes	10	1	3	Fair
Guelfi et al. (2013)	CD	NR	NR	NR	NR	Yes	NR	NR	Yes	NA	Yes	No	Yes	CD	4	1	9	Poor
Halliday et al. (2017)	Yes	NR	NR	NR	NR	Yes	No	Yes	No	NA	No	NR	Yes	Yes	5	3	6	Poor
Heiston et al. (2019)	Yes	Yes	NR	NR	NR	Yes	Yes	No	Yes	NA	No	No	Yes	No	6	4	4	Poor
Holliday et al. (2018)	Yes	Yes	Yes	NR	NR	CD	No	No	Yes	NA	No	Yes	Yes	No	6	4	4	Poor
Jakicic et al. (2011)	Yes	Yes	NR	NR	NR	Yes	No	Yes	CD	NA	No	Yes	Yes	Yes	7	2	5	Poor
Kirkwood et al. (2007)	Yes	NR	NR	NR	NR	Yes	No	NR	NR	NA	No	Yes	Yes	No	4	3	7	Poor
Macias-Cervantes et al. (2015)	Yes	Yes	Yes	NR	NR	No	No	NR	Yes	NA	CD	CD	Yes	No	5	3	6	Poor
Martin et al. (2019)	Yes	Yes	Yes	No	Yes	Yes	Yes	Yes	Yes	NA	Yes	Yes	Yes	No	11	2	1	Fair

Supporting Information - Beaulieu et al. Effect of Exercise Training Interventions on Energy Intake and Appetite Control in Adults with Overweight or Obesity: A Systematic Review and Meta-Analysis
k.beaulieu@leeds.ac.uk

References	Criteria														Total "Yes"	Total "No"	Total "other"	Quality rating
Martins et al. (2017)	Yes	NR	NR	NR	NR	Yes	No	No	Yes	NA	Yes	No	Yes	No	5	4	5	Poor
Nieman et al. (1990)	Yes	NR	NR	NR	NR	Yes	No	Yes	Yes	NA	No	NR	Yes	No	5	3	6	Poor
Quist et al. (2019)	Yes	Yes	NR	NR	NR	Yes	No	Yes	Yes	NA	Yes	No	Yes	No	7	3	4	Poor
Reseland et al. (2001)	Yes	NR	Yes	NR	NR	Yes	Yes	CD	CD	NA	No	No	Yes	No	5	3	6	Poor
Rhew et al. (2007)	Yes	NR	NR	NR	NR	Yes	Yes	Yes	Yes	NA	No	No	Yes	No	6	3	5	Poor
Riou et al. (2019)	Yes	NR	NR	NR	NR	Yes	Yes	NR	NR	NA	Yes	No	Yes	No	5	2	7	Fair
Rosenkilde et al. (2012)	Yes	Yes	NR	NR	NR	Yes	Yes	Yes	Yes	NA	Yes	Yes	Yes	No	9	1	4	Fair
Rosenkilde et al. (2013)	Yes	Yes	NR	NR	NR	Yes	Yes	Yes	Yes	NA	Yes	Yes	Yes	No	9	1	4	Fair
Sim et al. (2015)	Yes	Yes	NR	NR	NR	NR	Yes	Yes	Yes	NA	Yes	No	Yes	Yes	8	1	5	Good
Washburn et al. (2012)	Yes	NR	NR	NR	NR	Yes	Yes	Yes	Yes	NA	No	No	Yes	No	6	3	5	Poor
Washburn et al. (2015)	Yes	NR	NR	No	Yes	Yes	No	Yes	Yes	NA	Yes	No	Yes	No	7	4	3	Poor
Willis et al. (2019)	No	NR	NR	No	Yes	Yes	No	Yes	Yes	NA	Yes	No	No	No	5	6	3	Poor
Single-group interventions																		
Bryant et al. (2012)	Yes	Yes	Yes	CD	CD	Yes	Yes	No	CD	Yes	No	Yes	-	-	7	2	3	Poor
Caudwell et al. (2013a)	No	Yes	Yes	CD	CD	Yes	Yes	No	CD	Yes	No	Yes	-	-	6	3	3	Poor
Caudwell et al. (2013b)	Yes	Yes	Yes	CD	CD	Yes	Yes	No	CD	Yes	No	Yes	-	-	7	2	3	Poor
Cornier et al. (2012)	Yes	Yes	Yes	CD	CD	Yes	No	No	CD	Yes	Yes	Yes	-	-	7	2	3	Poor
Crampes et al. (2003)	Yes	Yes	Yes	CD	CD	Yes	No	No	CD	Yes	Yes	Yes	-	-	7	2	3	Poor
Garnier et al. (2015)	Yes	Yes	Yes	CD	CD	Yes	No	No	No	Yes	Yes	Yes	-	-	7	3	2	Poor
Halliday et al. (2014)	Yes	Yes	Yes	CD	CD	Yes	No	No	CD	Yes	Yes	Yes	-	-	7	2	3	Poor
Kanaley et al. (2014)	Yes	Yes	Yes	CD	CD	Yes	Yes	No	CD	Yes	No	Yes	-	-	7	2	3	Poor
King et al. (2008)	Yes	No	Yes	CD	CD	Yes	Yes	No	CD	Yes	No	Yes	-	-	6	3	3	Poor

Supporting Information - Beaulieu et al. Effect of Exercise Training Interventions on Energy Intake and Appetite Control in Adults with Overweight or Obesity: A Systematic Review and Meta-Analysis

k.beaulieu@leeds.ac.uk

References	Criteria														Total "Yes"	Total "No"	Total "other"	Quality rating
King et al. (2009)	Yes	Yes	Yes	CD	CD	Yes	Yes	No	No	Yes	No	Yes	-	-	7	3	2	Poor
Manthou et al. (2010)	Yes	Yes	Yes	CD	Yes	Yes	Yes	No	Yes	Yes	Yes	Yes	-	-	10	1	1	Good
Martins et al. (2010)	Yes	Yes	Yes	CD	CD	Yes	Yes	No	No	Yes	No	Yes	-	-	7	3	2	Poor
Martins et al. (2013)	Yes	Yes	Yes	CD	CD	Yes	Yes	No	No	Yes	No	Yes	-	-	7	3	2	Poor
Myers et al. (2019)	Yes	Yes	Yes	CD	CD	Yes	Yes	No	Yes	Yes	Yes	Yes	-	-	9	1	2	Fair
Woo et al. (1982a)	Yes	No	Yes	CD	No	Yes	Yes	No	Yes	Yes	Yes	Yes	-	-	8	3	1	Poor
Cross-over trials																		
Alkahtani et al. (2014)	No	NA	NA	NR	NR	Yes	NR	Yes	Yes	NR	Yes	No	Yes	CD	5	2	7	Poor
Damour et al. (2019)	Yes	NR	NR	NR	NR	No	NR	Yes	Yes	NA	No	No	Yes	CD	4	3	7	Poor
Woo et al. (1982b)	No	NA	NR	NR	NR	No	NR	No	CD	NA	Yes	No	Yes	CD	2	4	8	Poor

Criteria controlled trials: (1) Randomized study; (2) Adequate randomization method; (3) Treatment allocation concealment; (4) Blinding treatment assignment; (5) Blinding outcome assessors; (6) Similar baseline characteristics; (7) Drop-out rate <20%; (8) Differential drop-out rate between groups <15%; (9) High adherence; (10) Similar background treatments; (11) Valid and reliable outcome measures; (12) Sample size justification; (13) Pre-specified outcomes/subgroups; (14) All randomized participants analysed (ITT analysis).

Criteria single-group intervention studies: (1) Question/objective clearly stated; (2) Eligibility criteria pre-defined; (3) Representativeness; (4) Enrolment rates; (5) Sufficient sample size; (6) Intervention clearly described and delivered consistently (e.g. high adherence); (7) Valid and reliable outcome measures; (8) Outcome assessors blinded; (9) Drop-out rate <20% or intent to treat analysis; (10) Statistical analyses examined changes in outcomes; (11) Multiple time points for outcome measurement; (12) Account for individual changes.

Criteria cross-over trials: (1) Randomized study; (2) Adequate randomization method; (3) Order allocation concealment; (4) Blinding treatment order assignment; (5) Blinding outcome assessors; (6) Similar baseline characteristics; (7) Drop-out rate <20%; (8) Sufficient time frame for effect; (9) High adherence; (10) Similar background treatments; (11) Valid and reliable outcome measures; (12) Sample size justification; (13) Pre-specified outcomes/subgroups; (14) All randomized participants analysed (ITT analysis).

Shaded criteria represent fatal flaws if answered ‘No’ or ‘Other (CD, NA, NR)’.

NA, not applicable; NR, not reported; CD, cannot determine

Table S3. Findings of included studies

Reference	Findings	Study author's conclusion	Overview authors' assessment of conclusions	Energy intake	Appetite ratings	Eating behaviour traits or food reward
Randomized and non-randomized controlled trials						
Alizadeh et al. (2017)	- Energy (morning: -362 kcal, evening: -28 kcal), carbohydrate (morning: -50 g, evening: +7 g) and fat (morning: -11 g, evening: +12 g) intake change pattern difference between groups (group by time interactions p≤0.06).<br>- No between group differences in VAS changes pre and post exercise sessions (data not shown).	"[…] moderate-to-high intensity morning exercise could lead to significant changes in calorie intake and body composition over weeks, while exercising in the evening did not make such differences"	Agree that results demonstrate potential benefits of morning exercise on body weight and appetite control, but conclusions based on self-reported measures of energy intake.	Morning exercise: ↓ Evening exercise: ↔	↔	NR
Bales et al. (2012)	- Overall, energy intake decreased by 127 kcal[a] (p=0.003), mainly from fat (-4.5 g[a], p=0.026) and carbohydrate (-21.1 g[a], p=0.002). Percentage intake of each macronutrient did not change.<br>- By group, AT decreased by 117 kcal[a] (p=0.145), RT decreased by 179 kcal[a] (p=0.073) and AT+RT decreased by 185 kcal[a] (p=0.073).<br>- Fat intake (g) decreased in RT (-10 g[a], p=0.05) and AT+RT (-8.8 g[a], p=0.044) and % fat intake increased in AT (+1.2% kcal[a], p=0.02) and decreased in AT+RT (-1.3% kcal[a], p=0.039).	"We found that energy intakes did not increase in response to aerobic or resistance exercise' in fact, energy intakes decreased slightly between the beginning and the end of training. These results counter the claim that sedentary individuals who initiate a long-term exercise program increase their energy intakes in a compensatory fashion."	Appropriate conclusions based on available data, but important to take into account that conclusions are based on self-reported measures of energy intake.	↓	NR	NR

Reference	Findings	Study author's conclusion	Overview authors' assessment of conclusions	Energy intake	Appetite ratings	Eating behaviour traits or food reward
	- Protein intake (%) increased in AT+RT (+1.2% kcal[a], p=0.048).<br>- Carbohydrate intake (g) decreased in AT (-24.4 g[a], p=0.012) and AT+RT (-22 g[a], p=0.085) and % carbohydrate intake only decreased in AT (-2.3% kcal[a], p=0.044)					
Beaulieu et al. (2020)	- Exercise training led to reduction in wanting (-4.1 [95% CI -7.8, -0.4], p=0.03), binge eating score (-1.5 [95% CI -2.7, -0.4], p=0.01), and disinhibition (-0.7 [95% CI -1.3, -0.1], p=0.02).<br>- No exercise training effect on liking.	"[…] a 12-week exercise intervention reduced wanting scores for high-fat foods and trait binge eating relative to non-exercising Controls. A reduction in trait disinhibition with exercise was apparent but to a lesser degree"<br>"[...] it cannot be claimed that such an improvement will be seen in all people undertaking exercise"	Appropriate conclusions based on available data.	NR	NR	Wanting for high-fat food: ↓<br>Liking for high-fat food: ↔<br>Disinhibition & binge eating: ↓<br>Restraint and susceptibility to hunger: ↔
Bhutani et al. (2013)	- No changes in restrained eating, uncontrolled eating or emotional eating observed in EX.<br>- Greater changes in restrained eating were observed in ADF+EX and ADF (increase in both) relative to EX.<br>- Greater changes in uncontrolled eating and emotional eating observed in ADF+EX (decrease) relative to EX (no change).	"[…] the combination of ADF+EX increases restrained eating while decreasing uncontrolled and emotional eating. Taken together, endurance exercise is an excellent adjunct therapy to ADF, as it leads to positive behavioural changes that may contribute to long-term steady weight loss"	Exercise alone appeared to have minimal impact on self-reported energy intake and eating behaviour traits.	↔	NR	Restraint, uncontrolled eating, emotional eating: ↔

Reference	Findings	Study author's conclusion	Overview authors' assessment of conclusions	Energy intake	Appetite ratings	Eating behaviour traits or food reward
	- No changes in energy or macronutrient intake in EX and no differences in change scores across intervention groups.					
Brandon & Elliot-Loyd (2006)	African American exercisers:<br>- Total energy intake increased (p<0.05) from 1586 (151) kcal at baseline (wk 1) to 1830 (120) kcal post-training (wk 16).<br>- Carbohydrate intake (g) increased by 23 g from baseline to wk 9 (p<0.05), and a further 19 g post-training (p<0.05).<br>White exercisers:<br>- Energy intake decreased from 1693 (158) kcal at baseline to 1478 (155) kcal at wk 9 (p<0.05), and was not different to baseline post-training, (1606 (126) kcal).<br>- Carbohydrate intake decreased by 34 g from baseline to wk 9 (p<0.05), and was not different to baseline post-training (-21 g).<br>- Data for the controls NR.	"The walking dose did not produce a significant body composition response after the intervention for the [African American] women. However, the walking dose did allow the [African American exercisers] to maintain weight and percentage of fat even though they were consuming 16% more energy at the conclusion of the training intervention. […] Cultural and racial issues related to energy consumption patterns appear to be important when designing programs for [African American] women."	Appropriate conclusions based on available data, but important to take into account that conclusions are based on self-reported energy intake.	African American: ↑<br>White: ↔	NR	NR
Di Blasio et al. (2010)	- No change in whole day total energy or macronutrient intake.<br>- Evening exercise group increased % total energy intake consumed in the morning (6 (6) %,	"[…] major body composition improvement occurs when exercise is taken in conjunction with healthy dietary behaviour. In our case, the major	Conclusions too strongly worded for small effects and self-reported energy intake.	↔	NR	NR

Reference	Findings	Study author's conclusion	Overview authors' assessment of conclusions	Energy intake	Appetite ratings	Eating behaviour traits or food reward
	p=0.04) whereas no change observed in morning group.<br>- Morning group decreased % protein intake in the afternoon while it increased in the evening group (-2 (6) vs +4 (6) %, p=0.02).	change in [energy intake] daily distribution was linked to the [change of fat mass (kg)] and occurred in the [evening group]. Our hypothesis is that according to the time of day, walking could determine a difference dietary habit modification."				
Donnelly et al. (2003)	Males:<br>- No differences at baseline or at 16 months in intakes of energy, fat (g) or carbohydrate (g) between exercise and control groups.<br>- Protein intake increased in exercisers from 102 (23) g at baseline to 106 (27) g at 16 months (p<0.05) but no change when expressed in % daily energy intake.<br>Females:<br>- No differences between exercisers or controls.	"[...] we conclude that moderately intense exercise is not likely to change the macronutrient content of an ad libitum diet (ie, increase in carbohydrate intake and decrease in fat intake) in the absence of dietary advice."	Appropriate conclusions based on available data.	↔	NR	NR
Dorling et al. (2019)	- No exercise effects on food intake at buffet meal.<br>- Prospective food consumption before lunch increased in the controls relative to 20 KKW (7 (3); p=0.03, main effect of group).	"We additionally showed that appetitive measures, anthropometry, and body composition were not appreciably affected by the interaction between race and exercise."	Appropriate conclusions based on available data.	↔	↔	NR

Reference	Findings	Study author's conclusion	Overview authors' assessment of conclusions	Energy intake	Appetite ratings	Eating behaviour traits or food reward
Flack et al. (2018)	- Change scores for % fat intake differed between groups (1500 kcal/wk: +3.0 [95% CI 0.3, 5.6] % vs 3000 kcal/wk: -2.2 [-4.7, 0.4] %, p=0.01)<br>- Both groups similarly reduced their reinforcing value of a snack foods (p=0.01); however, when desire to eat was controlled for, food reinforcement results did not change.<br>1500 kcal/wk group:<br>- Increase in % fat intake (+3% [95%CI 0.3, 5.6], p=0.03)<br>- Decrease in % carbohydrate intake (-4.1% [95%CI -8.1, -0.2], p=0.04).<br>3000 kcal/wk group:<br>- Decrease in energy intake (-324 kcal [95%CI -603, -45], p=0.03) in 3000 kcal group only.<br>- Increase in % protein intake (+2.4% [95%CI 0.2, 4.7], p=0.04).	"[…] participants may have been less motivated to consumed less-healthy snack foods upon completion of the exercise intervention. Indeed, engagement in an exercise program can exert a 'spillover' effect, resulting in other health behaviour changes, including dietary modification […]"	Appropriate conclusions based on available data.	1500 kcal/wk: ↔<br>3000 kcal/wk: ↓	NR	Reinforcing value of food: ↓ (when controlling for desire to eat: ↔)
Foster-Schubert et al. (2012)	- In exercisers, energy intake decreased by 9%, with no difference in changes across groups (diet: -13%, diet+exercise: -14%, control: -11%).<br>- No change in fat intake in exercisers (-3%), whereas diet and diet+exercise groups decreased fat	*Outcomes not discussed.*	Exercise alone appeared to have minimal impact on self-reported energy intake.	↔ relative to other interventions	NR	NR

Reference	Findings	Study author's conclusion	Overview authors' assessment of conclusions	Energy intake	Appetite ratings	Eating behaviour traits or food reward
	intake by 18% and 20%, respectively (control: -6%).					
Guelfi et al. (2013)	- Increase in fasting (+15 mm, p=0.001) and postprandial fullness (+14 mm, p=0.006) following aerobic training only.<br>- No change in fasting or postprandial hunger.	"[…] 12 weeks of regular aerobic exercise training stimulates satiety in both the fasting state and for up to 2 h postprandial, while an equivalent period of resistance training does not."	Appropriate conclusions based on available data.	NR	Hunger (fasting and postprandial): ↔<br>Fullness (fasting and postprandial): ↑ (aerobic only)	NR
Halliday et al. (2017)	- Energy intake decreased by 76 kcal from baseline to month 9 (p<0.05) and 67 kcal from baseline to month 15 (p<0.05).<br>- Reduction in carbohydrate intake (g) from baseline to month 3, 9 and 15 (all p<0.05).<br>- No change in overall diet quality across the intervention.<br>- From baseline to 3 months, HEI-2010 scores for whole fruit, whole grains and refined grains decreased (p<0.01). Whole fruit and refined grain scores returned to baseline during maintenance phase.<br>- Total vegetable and greens and beans score increased (p<0.002) during maintenance phase (month 3 to 9), but returned to baseline during no-contact phase.	"[…] [resistance training] may be a unique mode of [physical activity] in its ability to influence […] dietary intake among previously inactive, overweight/obese adults with prediabetes."	Conclusions based on self-reported data and small effects.	↓	NR	NR

Reference	Findings	Study author's conclusion	Overview authors' assessment of conclusions	Energy intake	Appetite ratings	Eating behaviour traits or food reward
Heiston et al. (2019)	- No change in fasting or 120-min hunger.<br>- No change in fasting fullness but fullness at 120 min decreased after both interventions (continuous: -2.1 mm, HIIT: -11 mm; main effect p=0.05).<br>- Reduction in energy intake (continuous: 173 kcal, HIIT: 285 kcal, main effect p=0.09) and protein intake (continuous: 10 g, HIIT: 10 g, main effect p=0.05).	"[…] exercise tended to reduce total energy and protein intake independent of intensity, suggesting that training prevented compensatory weight gain. […] Further research is required to better understand how these outcomes of appetite are affected by long-term exercise […]"	Appropriate conclusions based on available data and small effects, but important to take into account that conclusions are based on self-reported energy intake and a 2-wk intervention.	↓	Hunger (fasting and postprandial): ↔<br>Fasting fullness: ↔<br>Postprandial fullness: ↓	NR
Holliday et al. (2018)	- Energy intake decreased more in points-based (-445 kcal) and control (-233 kcal) groups than structured exercise group (-52 kcal; interaction p=0.054).<br>- Fat intake decreased by 19 g [95% CI -32.2, -5.8] in points-based group (p=0.006) and was ~15 g lower than structured exercise group at 24 wk (p=0.035). No change in % macronutrient intake.	"[Modest but meaningful reductions in body weight and body fat with a points-based approach to physical activity] is likely a result of modest reductions in sedentary time, increases in light activity and a spill-over effect of altered eating behaviour and reduced energy intake."	Appropriate conclusions based on available data, but important to take into account that conclusions are based on self-reported energy intake.	Points-based physical activity: ↓<br>Structured exercise: ↔	NR	NR
Jakicic et al. (2011)	- No group by time interaction on changes in energy intake (-201 (517) kcal or intake of fat (-0.7 (6.7) %), carbohydrate (+0.6 (7.0) %) or protein (+0.2 (2.6) %).<br>- Eating behaviour score improved post-intervention (from	"[Individuals who lose weight with physical activity] may also self-select to change their eating behaviors, which in combination with physical activity, resulted in the	Conclusions ambiguous - results do not show consistent effects for eating behaviours and energy intake	↔	NR	Overall eating behaviours: ↑ (improvement)<br>Weight losers > weight

Reference	Findings	Study author's conclusion	Overview authors' assessment of conclusions	Energy intake	Appetite ratings	Eating behaviour traits or food reward
	73.1 (9.9) to 78.3 (10.8), p<0.001) but no differences between groups.<br>- No differences in energy or macronutrient intake across weight loss groups.<br>- Weight loss group × time interaction (p<0.001) for change in eating behavior score, with weight losers (10.0 (9.9)) increasing more than weight maintainers (2.8 (6.8)) and gainers (4.9 (10.4)).	significant decrease in weight."	(energy intake is self-reported).			maintainers & weight gainers
Kirkwood et al. (2007)	- In physical activity group, energy intake only decreased at wk 6 by 213 kcal/d (p<0.05) (energy intake also decreased in control group relative to baseline, by 357 kcal at wk 6 and 371 kcal wk 12, p<0.05).<br>- Greater reduction in energy intake in the diet+activity group (-693 kcal) relative to activity only (-177 kcal; p<0.001).<br>- Greater change in % fat intake activity group (+4%) compared to diet or diet+activity (both -5%, respectively, p<0.01).<br>- Increase in carbohydrate in diet+activity group (+2%) compared to decrease in activity alone (-4%; p<0.05).<br>- Greater change in protein intake in diet and diet+activity	"This highlights the need for dietary advice to accompany advice on increasing activity to achieve weight loss."	Conclusions weak as based on unsupervised exercise intervention and self-reported energy intake.	↔	NR	NR

Reference	Findings	Study author's conclusion	Overview authors' assessment of conclusions	Energy intake	Appetite ratings	Eating behaviour traits or food reward
	groups (both +3%) compared to activity only (-0.3%, p<0.05)					
Macias-Cervantes et al. (2015)	- No change in energy intake in response to exercise (-43 kcal, p=0.737) compared to decrease in both diet groups (~650-700 kcal, p<0.001).	"[…] exercise alone did not have significant metabolic effects in our study […]."	Exercise alone appeared to have minimal impact on self-reported energy intake.	↔	NR	NR
Martin et al. (2019)	- Adjusted DLW energy intake increased in both exercise groups relative to control group (8 KKW: 91 kcal [95%CI 35, 146]; 20 KKW: 124 kcal [95%CI 65, 183]; control: -2.3 kcal [95%CI -58, 54]; p<0.01).<br>- No changes in test meal energy intake (lunch and dinner combined) or appetite sensations.<br>- 8 KKW (0.2 [95% CI -0.2, 0.6]) increased preference for high-fat/high-carbohydrate foods compared to 20 KKW (-0.5 [95% CI -1.0, -0.1]; p=0.04)<br>- Compensators had increases in adjusted DLW energy intake (C: 149 kcal [95%CI 91, 208]; NC: 60 kcal [95%CI 1, 120], p=0.03), cravings for sweets (C: 0.3 [95%CI 0.1, 0.6]; NC: -0.2 [95%CI -0.4, 0.1]; p=0.01), and retrospective hunger (C: 5 [95%CI 0.1, 10.1]; NC: -2.3 [95%CI -7.4, 2.8]; p=0.03) and prospective	"[Body weight compensation in response to an exercise intervention] is due to increased energy intake […] and increased cravings for sweets, hunger, prospective food consumption, and compensatory health beliefs (and a smaller reduction in disinhibition). The results suggest that compensators might 1) differ in important ways prior to exercise that contribute to a differential response, 2) not experience some physiological (e.g., increased cardiorespiratory fitness) benefits of exercise that lead to exercise being more difficult/aversive, 3) have different psychological responses to exercise (e.g.,	Appropriate conclusions based on available data.	All exercisers: ↑<br>Compensators > non-compensators	Compensators: ↑	Restraint, disinhibition, susceptibility to hunger: ↔<br>Preference for high-fat/high-charbohydrate food: 8 KKW (↑) > 20 KKW (↓)<br>Compensators: ↑ eating behaviours promoting overconsumption

Reference	Findings	Study author's conclusion	Overview authors' assessment of conclusions	Energy intake	Appetite ratings	Eating behaviour traits or food reward
	food consumption (C: 2 [95%CI -1.9, 6.7]; NC: -3.8 [95%CI -8.2, 0.5]; p=0.03).<br>- Compensators had reductions in preferences for high carbohydrate foods (C: -0.4 [95%CI -0.8, 0.1]; NC: 0.3 [95%CI -0.2, 0.7]; p=0.03), low-fat foods (C: -0.4 [95%CI -0.8, 0.0]; NC: 0.3 [95%CI -0.2, 0.7]; p=0.02) and low-fat/high-carbohydrate foods (C: -0.4 [95%CI -0.8, 0.1]; NC: 0.4 [95%CI -0.1, 0.9]; p=0.01) and smaller reductions in disinhibition (C: -0.3 [95%CI -0.9, 0.4]; NC: -1.3 [95%CI -1.9, -0.6]; p=0.02).	increased compensatory beliefs and smaller reductions in disinhibition) that affect other health behaviours, and 4) experience an increased drive to eat."				
Martins et al. (2017)	- Increase in fasting (+1 cm; p=0.01) and 3-h AUC hunger (+69 mm*min, ~16%, p=0.048) post-training in all groups.<br>- No changes in food reward post-training.	"[…] the impact of MICT vs HIIT on appetite, in obese previously sedentary individuals, does not seem to differ. Neither exercise modality seems to induce meaningful changes in either subjective or objective appetite measures or food hedonics, at least when weight loss is minimal."	Appropriate conclusions based on available data. Perhaps a larger dose of exercise is required to see effects.	NR	Hunger (fasting and postprandial): ↑<br>Fullness (fasting and postprandial): ↔	Liking and wanting: ↔
Nieman et al. (1990)	- Exercise group decreased energy intake by 9% (p<0.05), whereas the control group increased	"[…] obese persons tend to maintain or decrease their energy intake [with increase	Appropriate conclusions based on available data	↓	NR	NR

Reference	Findings	Study author's conclusion	Overview authors' assessment of conclusions	Energy intake	Appetite ratings	Eating behaviour traits or food reward
	intake by 9% (between group p=0.09).<br>- Carbohydrate intake (g) decreased by 23 g in exercise group and increased by 19 g in control group (interaction p=0.013)<br>- Difference in the change in number of bread/cereal exchanges across groups (p<0.006), exercise group decreased by 1.6 exchange whereas control group increased by 0.9 exchange.	energy expenditure]. Thus exercise training by the obese can help prevent weight gain and should, in the long term, lead to a negative energy balance and promote weight loss."	and small effects, but important to take into account that conclusions are based on self-reported energy intake.			
Quist et al. (2019)	- At 3 months, test meal energy intake was lower in vigorous relative to control (-280 kcal, p<0.01). At 6 months, test meal energy intake was lower in vigorous relative to moderate (-158 kcal, p=0.03). No differences found for cumulative daily energy intake.<br>- At 6 months, fasting hunger was higher in bike relative to vigorous (+13 mm, p=0.03)<br>- At 3 months, postprandial prospective food consumption was lower in moderate (-19%, p=0.02) and vigorous (-30%, p<0.001) relative to control, and in vigorous relative to bike (p<0.01). Hunger was lower in moderate (p=0.04) and vigorous (p<0.01) compared to bike. At 6	"In summary, 6 mo of active commuting and leisure-time exercise of different intensities did not increase markers of appetite in women and men with overweight and obesity. […] Furthermore, our findings suggest that exercise needs to be of vigorous intensity to affect appetite in individuals with overweight and obesity."	Appropriate conclusions based on available data.	Test meal: ↑ moderate-intensity > vigorous-intensity<br>Total daily: ↔	Fasting hunger: ↑ bike > vigorous-intensity	Restraint & disinhibition: ↔<br>Susceptibility to hunger: ↓ bike & vigorous-intensity

Reference	Findings	Study author's conclusion	Overview authors' assessment of conclusions	Energy intake	Appetite ratings	Eating behaviour traits or food reward
	months, prospective food consumption was still lower in vigorous compared with bike (p=0.04) and moderate (p=0.048).<br>- No change in restraint and disinhibition observed. At 6 months, susceptibility to hunger decreased in bike and vigorous compared to moderate (p<0.03) but not control.					
Reseland et al. (2001)	- Relative to diet groups, exercise only and control groups did not change energy intake over the intervention.<br>- Fat intake (%) decreased in exercise only group (-1.6%) whereas it decreased by ~5% in both diet groups (p≤0.001).	*Outcomes not discussed.*	Exercise alone appeared to have minimal impact on self-reported energy intake.	↔	NR	NR
Rhew et al. (2007)	- Changes in energy intake not different between groups.<br>- Fat intake decreased by 1.1 g (-1.4% total energy intake) in the exercise group and increased by 1.7 g (+0.7% total energy intake) in the control group from baseline to 3 months (interaction p=0.04).	"[…] exercise does not appear to induce substantial changes in other health behaviours in previously sedentary, overweight/obese women participating in an exercise intervention trial."	Appropriate conclusions based on available data.	↔	NR	NR
Riou et al. (2019)	- No changes in energy intake observed in response to the training interventions.<br>- Trend for an increase in prospective food consumption after lunch observed across both exercise	"It would seem logical to conclude that the compensation from exercise-induced [energy expenditure] was not mediated by variations	Conclusions difficult to understand from the outcomes reported.	↔	Prospective food consumption: ↑	Susceptibility to hunger: ↑ Wanting for high-fat food: ↓

Reference	Findings	Study author's conclusion	Overview authors' assessment of conclusions	Energy intake	Appetite ratings	Eating behaviour traits or food reward
	groups (wk -4: 4 (5) mm, wk 1: 6 (7) mm, wk 12-14: 13 (22), p=0.08).<br>- Susceptibility to hunger increased in both exercise groups from wk -4 to wk 12 (data not shown, p=0.004).<br>- Wanting for fat decreased across both exercise groups from wk 1 to wk 12-14 (data not shown; p=0.03).<br>- Increase in liking for savoury foods pre- to post-acute exercise at wk 1 but decrease at wk 12-14 (data not shown; interaction p=0.002).	[energy intake]. […] This means that changes in [energy expenditure] cannot by themselves explain the changes in body energy stores that were measured, even if [non-structured physical activity] was significantly reduced for both groups."				Liking for savoury foods in response to acute exercise: ↓
Rosenkilde et al. (2012)	- No difference in habitual energy intake in response to the intervention between or within groups (control: 0 kcal, moderate: +20 kcal, high: +130 kcal).<br>- No change in % macronutrient intake between or within the groups.<br>- No changes between or within groups in high-carbohydrate or low-carbohydrate energy intake.	"[…] we propose that the introduction of a moderate dose of exercise may actually lead to an increase in [nonexercise activity thermogenesis] without any increase in [energy intake] resulting in a "bonus effect," whereas a higher dose of exercise may lead to an increase in [energy intake] and, thereby, a degree of compensation and less than expected loss of [fat mass]."	Conclusions do not follow from the results reported.	↔	NR	NR

k.beaulieu@leeds.ac.uk

Reference	Findings	Study author's conclusion	Overview authors' assessment of conclusions	Energy intake	Appetite ratings	Eating behaviour traits or food reward
Rosenkilde et al. (2013)	- Fasting fullness (p<0.001) and satiety (p=0.03) increased in high-dose (fullness: 36 (17) mm, satiety: 35 (15) mm) compared to both moderate-dose (fullness: 20 (12) mm, satiety: 28 (16) mm) and control (fullness: 21 (11) mm, satiety: 27 (13) mm)<br>- Compared to control, moderate-dose had lower susceptibility to hunger (-1.6 [95%CI -3.2, 0.0], p=0.06). No differences/changes in other eating behaviour traits.<br>- Postprandial fullness increased in high-dose compared to control (estimate: 0.163 [CI: 0.081, 0.245], p<0.001)<br>- Hunger was 34–50% lower 30 and 60 min after the breakfast in both moderate- and high-dose compared with control (p<0.05)<br>- Prospective food consumption reduced (27–33%) in high-dose at 30 and 60 min compared with control (p<0.012)<br>- No difference in energy intake, although removal of 3 outliers showed a tendency toward a decrease in EI in moderate compared	"In conclusion, overweight, previously sedentary men's participation in 12 wk of 30 or 60 min of daily endurance exercise did not result in signs of increased appetite despite the presence of a negative energy balance. Instead, fasting and postprandial perceptions of satiety and fullness […] increased after a high dose of daily endurance exercise. This short-term effect on markers of appetite does not elude why energy balance was equally negative between [moderate-dose] and [high-dose]."	Appropriate conclusions based on available data.	↔	Fasting fullness & satiety:<br>↑ high-dose<br>↔ moderate-dose<br>Postprandial hunger: ↓ moderate-dose & high-dose	↔

Reference	Findings	Study author's conclusion	Overview authors' assessment of conclusions	Energy intake	Appetite ratings	Eating behaviour traits or food reward
	with control (-115 kcal [CI: -244, 13]; p<0.08).					
Sim et al. (2015)	- Tendency for a reduction in test meal energy intake after high-energy preload relative to low-energy preload post-training in HIIT group only (-516 kJ [95%CI -271, -762]).<br>- No change in cumulative energy intake.<br>- No change in appetite ratings.	"In summary, we found that HIIT resulted in clinically meaningful improvements in appetite regulation whereas an equivalent period of MICT and [control] did not."	Conclusions based on small effects and sample size.	Improvement in acute (1 meal) energy compensation	↔	NR
Washburn et al. (2012)	- No differences in dietary intake during the intervention between groups (mean intake ~2200 kcal).	"[…] a 1-set [resistance training] program may be beneficial for weight maintenance in this population."	Appropriate conclusions based on available data.	↔	NR	NR
Washburn et al. (2015)	- In total sample, no significant change in absolute energy intake from baseline to post-intervention but energy intake relative to body weight increased in the 600 kcal group (32.3 (6.3) kcal/kg/day to 35.4 (8.2) kcal/kg/day and was unchanged in the 400 kcal and control groups from baseline to 10 months (group-time interaction p<0.01).<br>- In women, absolute energy intake was greater in the 600	"In summary, we found no significant change in energy or macronutrient intake in response to a 10-month supervised exercise program in overweight and obese young adults. The possibility of a threshold beyond which further increases in [exercise energy expenditure] do not produce a more negative	Appropriate conclusions based on available data.	↔	NR	NR

Reference	Findings	Study author's conclusion	Overview authors' assessment of conclusions	Energy intake	Appetite ratings	Eating behaviour traits or food reward
	kcal/session group compared with controls at 3.5 months (+484 kcal, p=0.036) and 7 months (+624 kcal, p=0.016)<br>- No effect of training on diet quality.	energy balance and potential sex differences in the energy intake response to increased levels of [exercise energy expenditure] observed in this study are potentially important […]."				
Willis et al. (2019)	- No between group differences in energy or macronutrient (g) intake. Tendency for Late Exercisers to have greater energy intake (+121 kcal) than other groups (early: -63 kcal; sporadic: -117 kcal).<br>- Relative energy intake (kcal/kg/day) increased across all groups (p=0.003), and percentage of energy intake as fat decreased across all groups (p=0.012).	"[…] engaging in morning exercise may result in more weight loss compared to engaging in a similar amount of exercise later in the day. […] individuals who performed most of their exercise sessions in the afternoon or evening tended to have slightly higher levels of [energy intake] and reduced [non-exercise physical activity and energy expenditure], suggesting that there are potentially important differences in the components of energy balance based on time of day exercise is performed."	Appropriate conclusions based on available data, but based on secondary data analysis; therefore, not an authentic RCT.	Morning exercise < Late exercise	NR	NR

Reference	Findings	Study author's conclusion	Overview authors' assessment of conclusions	Energy intake	Appetite ratings	Eating behaviour traits or food reward
Single-group interventions						
Bryant et al. (2012)	- No change in 24-h energy intake or susceptibility to hunger.<br>- Significant reduction in disinhibition (-17%, p<0.001) and increase (+20%, p<0.001) in restraint after training.<br>- Group-time interaction (p<0.05) showed that responders had a greater increase in restraint (+2.4, p<0.05) relative to non-responders (+0.7). Responders had greater restraint overall (p<0.01).	"[...] a decrease in disinhibition combined with an increase in restraint is a predictor of successful weight loss and other anthropometric markers [with exercise training]."	Appropriate conclusions based on available data.	↔	NR	Disinhibition: ↓<br>Restraint: ↑<br>Responders > Non-responders
Caudwell et al. (2013a)	- Significant main effect of training on high-energy density meal size (7.04 (2.49) to 6.80 (3.26) MJ, p<0.005) but not low-energy density meal size (4.60 (1.85) to 4.28 (1.76) MJ).<br>- No main effect of training on daily energy intake under each dietary condition.	*Outcomes not discussed.*	Exercise appeared to have reduced intake at a high-energy-density test meal, but this did not impact total daily energy intake.	Meal size:<br>High-energy-density ↓<br>Low-energy-density ↔<br>Total daily: ↔	NR	NR
Caudwell et al. (2013b)	- No changes in 24-h energy intake with training for either males or females.<br>- Increase in fasting hunger (males: 11 (4) mm, females: 14 (3) mm; p<0.0001) but no change in daily hunger area under the curve.	"[...] when exercise is closely monitored so that the [energy expenditure] for males and females is similar, there are no sex-based differences in the compensatory response to exercise."	Appropriate conclusions based on available data.	↔	Fasting hunger: ↑<br>Daily hunger: ↔<br>Satiety quotient: ↑	NR

k.beaulieu@leeds.ac.uk

Reference	Findings	Study author's conclusion	Overview authors' assessment of conclusions	Energy intake	Appetite ratings	Eating behaviour traits or food reward
	- SQ greater post-training (p<0.0001), and greater in females than males before and after the intervention (p=0.014).					
Cornier et al. (2012)	- No changes in eating behaviour traits or post-prandial appetite ratings.<br>- Reduced neuronal responses to visual food cues after intervention in bilateral parietal cortices, left insula and visual cortex (p<0.01).<br>- No change in the acute exercise response with exercise training.<br>- Self-reported energy intake decreased after training (2192 (208) to 1980 (159) kcal/d, p=0.049) but no change in macronutrient intake.	"Measures of eating-related behaviors and appetite, however, were not affected by the exercise intervention even despite modest weight/fat loss, suggesting that exercise may attenuate the changes in ingestive behavior expected with negative energy balance."	Appropriate conclusions based on available data, but important to take into account that conclusions are based on self-reported energy intake.	↓	↔	Eating behaviour traits: ↔<br>Neuronal response to food cues: ↓
Crampes et al. (2003)	- No change in energy or macronutrient intake pre to post-intervention.	*Outcomes not discussed.*	Exercise appeared to have minimal impact on self-reported energy intake.	↔	NR	NR
Garnier et al. (2015)	- No change in total energy intake post-intervention.<br>- % energy from carbohydrate decreased (-1.0 (6.2) %) and % energy from protein	"The main findings of our study indicate that although our walking program changed food consumption patterns, it did not have much impact on energy	Appropriate conclusions based on available data, but important to take into account that conclusions	↔	NR	NR

k.beaulieu@leeds.ac.uk

Reference	Findings	Study author's conclusion	Overview authors' assessment of conclusions	Energy intake	Appetite ratings	Eating behaviour traits or food reward
	increased (+0.7 (3.7) %) after training (p=0.05).<br>- Intake of fruit (-0.18 (1.0) portions/d; p<0.05) and sweet and fatty foods (-0.23 (1.1) portions/d; p<0.01) decreased, and intake of oils (+0.32 (1.0) portions/ d; p<0.0001) increased post-intervention.<br>- Women with the highest decrease in body weight had the most reduced consumption of fruits (–0.43 (0.16) portions/d; p<0.05), sugar and sweet foods (–0.098 (0.22) portions/d; p≤0.05) and fatty foods (–0.31 (0.21) portions/d; p<0.05).<br>- Women with the greatest reduction in fat mass had the highest decrease in fatty foods consumption (–0.20 (0.19) portions/d; p<0.05).	intake, thus suggesting that body weight loss was primarily due to energy output.”	are based on self-reported energy intake.			
Halliday et al. (2014)	- Energy intake decreased by 80 kcal (p<0.05) post-training.<br>- Reduction in carbohydrate intake (-10 g, p<0.05), but not change in % carbohydrate intake.<br>- Fruit and vegetable intake (excluding juice) decreased from 4.6 (2.3) to 4.1 (2.3) portions/d (p<0.05).<br>- Sweets/dessert intake decreased from 1.1 (0.8) to 0.9 (0.8) portions/d (p<0.05).	"Previously sedentary, prediabetic individuals who completed 12 weeks of resistance training without receiving dietary counselling reported decreasing total energy and carbohydrate (g) intake. Reduction in [fruits/vegetables] and sweets/desserts likely explains [this reduction]”.	Appropriate conclusions based on available data and small effects, but important to take into account that conclusions are based on self-reported energy intake.	↓	NR	NR

Reference	Findings	Study author's conclusion	Overview authors' assessment of conclusions	Energy intake	Appetite ratings	Eating behaviour traits or food reward
Kanaley et al. (2014)	- No difference in hunger or fullness pre- to post-training.	"[…] short-term aerobic exercise training may induce changes in [appetite-related peptide concentrations] before changes in hunger and fullness occur."	Appropriate conclusions based on available data and 15-day intervention.	NR	↔	NR
King et al. (2008)	- No significant changes in 24-h energy intake in pooled data with training, however non-compensators decreased energy intake by 130 (485) kcal/day, whereas compensators increased energy intake by 268 (455) kcal /day ($p<0.05$).<br>- In compensators, fat intake increased from 32.6 (3.5) to 34.9 (3.6) % ($p<0.05$).<br>- Compensators had greater hunger profile post-training than non-compensators.	"The identification and characterization of the various compensatory responses to exercise are useful for explaining the variability [in body weight loss] and could be used to improve the effectiveness of exercise."	Appropriate conclusions based on available data.	Overall: ↔<br>Non-compensators: ↓<br>Compensators: ↑	Daily hunger compensators > non-compensators	NR
King et al. (2009)	- Non-responders and responders had significantly greater fasting hunger (+24 and +38% change, respectively, $p<0.001$) but also had a greater satiety quotient post-training<br>- Only non-responders increased daily motivation to eat post-training (greater hunger, desire to eat and lower fullness, +43, +6 and -10%, respectively; $p<0.005$).	"[…] the effect of exercise on appetite regulation involves at least 2 processes: an increase in the overall (orexigenic) drive to eat and a concomitant increase in the satiating efficiency of a fixed meal."	Appropriate conclusions based on available data.	Responders: ↓<br>Non-responders: ↑	Fasting hunger: non-responders & responders ↑<br>Daily hunger: non-responders ↑<br>Satiety quotient: non-responders & responders ↑	NR

Reference	Findings	Study author's conclusion	Overview authors' assessment of conclusions	Energy intake	Appetite ratings	Eating behaviour traits or food reward
	- Positive association between changes in daily energy intake and change in body weight (p =0.26, p<0.05)<br>- Daily energy intake of the non-responders increased (+164 kcal/d), but no change (slight decline) in the energy intake of the responders (-126 kcal/d).					
Manthou et al. (2010)	- Overall, exercise increased energy intake by 10% (+234 kcal, p<0.05), with no difference between responders (+206 kcal) and non-responders (+246 kcal).	"[…] our data confirm that there is a large degree of interindividual variability in body fat loss in response to an exercise training intervention and indicated that, in overweight women, compensatory reductions in [energy expenditure] of physical activity outside exercise intervention can contribute to the failure of exercise to successfully induce fat loss."	Conclusions based on self-reported energy intake (which did not differ between groups), and it is hypothesized that only compensatory adaptations in energy expenditure lead to differences in body fat loss in response to exercise.	↑ in both responders and non-responders	NR	NR
Martins et al. (2010)	- There was an increase in fasted hunger (4.1 (1.6) to 6.5 (2.5) cm; p<0.01), desire to eat (4.8 (1.5) to 6.4 (2.3) cm; p<0.05) and prospective food consumption (6.0 (1.6) to 7.1 (2.0) cm; p<0.05), and a	“We can conclude that although exercise-induced weight loss leads to an increase in fasting [acylated ghrelin] and hunger sensations, […] exercise appears to balance this	Conclusions based on small effects.	NR	Fasting appetite: ↑<br>Postprandial hunger: ↑	NR

Reference	Findings	Study author's conclusion	Overview authors' assessment of conclusions	Energy intake	Appetite ratings	Eating behaviour traits or food reward
	reduction in fullness (3.5 (1.1) to 1.9 (1.5) cm; p<0.01) post-training.<br>- Greater postprandial hunger (p<0.01) and desire to eat (p<0.05) post-training.<br>- Week by time interaction for postprandial fullness (p<0.01; post hoc comparisons not shown).	increased orexigenic drive by improving the satiety response to a meal and the sensitivity of the appetite control system."				
Martins et al. (2013)	- No change in test meal energy intake.<br>- Exercise-preload interaction (p=0.011) showing that cumulative energy intake after high-energy preload was higher than after the low-energy preload at baseline (2118 (775) vs 1803 (421) kcal, p=0.001), whereas post-training, cumulative energy intake after the high-energy preload was lower than after the low-energy preload (1799 (649) vs 2044 (763) kcal, p=0.001)<br>- No change in appetite ratings.	"The results of the present study showing […] a better compensatory response to an energy preload extend and strengthen our previous findings."	Conclusions based on combination of test meal and self-report data.	Improvement in cumulative energy compensation	↔	NR
Myers et al. (2019)	- Total and ad libitum energy intake increased by 178 (372) kcal (p=0.028) after training (driven by increase of 108 (255) kcal in snack box energy intake (p=0.048)).<br>- No significant change in fasting hunger but daily hunger	"Despite finding a short-term increase in energy intake during laboratory probe<br>days, the magnitude of this effect was not sufficient to fully explain the difference	Appropriate conclusions based on available data.	↑	Fasting appetite: ↔<br>Daily appetite: ↑	NR

Reference	Findings	Study author's conclusion	Overview authors' assessment of conclusions	Energy intake	Appetite ratings	Eating behaviour traits or food reward
	increased after training (22 (17) to 26 (16) mm; p=0.01).<br>- No significant change in fasting fullness but daily fullness decreased after training (60 (20) to 56 (20) mm; p=0.03).	between predicted and observed weight loss."				
Woo et al. (1982a)	- No changes in energy intake over the 57 days.	"The combination of moderate exercise with energy intake at the level self-selected in this study produced a negative energy balance. Therefore, exercise, if combined with a palatable but not gourmet diet, can be useful treatment for weight reduction."	Appropriate conclusions based on available data, but based on very small sample size.	↔	NR	NR
Crossover trials						
Alkahtani et al. (2014)	- Tendency for suppression of desire to eat after acute exercise post-training with HIIT (-16 (22) mm) compared to MIIT (+4 (27) mm; interaction p=0.07).<br>- Tendency for increase with MIIT (+6 (20) mm) and decrease with HIIT (-13 (16) mm) in explicit liking for high-fat non-sweet foods after acute exercise post-training (interaction p=0.09).<br>- No effects of training on food intake or energy intake.	"The tendencies of eating behaviour including medium term-Ex hunger and desire to eat, medium term-Ex explicit liking for energy-dense food, medium-term constant-load exercise-induced food and fat intake collectively suggested that HIIT is a better strategy than MIIT to minimize the compensation of eating	Conclusions based on very small sample and effects.	↔	MIIT > HIIT	Liking high-fat non-sweet food: MIIT > HIIT

Reference	Findings	Study author's conclusion	Overview authors' assessment of conclusions	Energy intake	Appetite ratings	Eating behaviour traits or food reward
	- Tendency for fat intake (g) to increase after MIIT (+14 g) and decrease after HIIT (-7 g; interaction p=0.07).	behaviour during interval training among males."				
Damour et al. (2019)	- Energy (-1153 kcal), protein (-42 g), fat (-65 g) and carbohydrate (-151 g) decreased across both interventions (p<0.02).<br>- Reduction in protein intake after Ex (-1.5 %) compared to a maintenance in ExMeal (+0.4%; interaction p=0.05).<br>- Number of consumed portions of dairy and alternatives (-1.6 portions/d), vegetables (-1.3 portions/d), meat and alternatives (-1.4 portions/d), and grains (-3.8 portions/d) decreased during both interventions (p<0.02).	"[…] both programs (ExMeal and Ex) yielded positive but similar benefits in anthropometric variables, total energy, macronutrient and food-group intakes. […] ExMeal resulted in 2% more energy obtained from protein compared to Ex […]"	Conclusions based on very small sample and effects.	↓	NR	NR
Woo et al. (1982b)	- Daily energy intake was no different between the three experimental treatments.	"Since no hyperphagic response occurred, negative balance during exercise balance was obtained. […] Therefore, the effect of exercise alone in the treatment of obesity will be tempered by the dietary milieu in which it is imposed."	Appropriate conclusions based on available data, but based on very small sample size.	↔	NR	NR

Supporting Information - Beaulieu et al. Effect of Exercise Training Interventions on Energy Intake and Appetite Control in Adults with Overweight or Obesity: A Systematic Review and Meta-Analysis
k.beaulieu@leeds.ac.uk

Values are means (SD), unless specified otherwise. [a]Median (interquartile range/25th, 75th quartile). ADF, alternate day fasting; AT, aerobic training; C, compensators; DLW, doubly-labelled water; EX, exercise; HIIT, high-intensity interval exercise; KKW, kcal/kg body weight/week; MICT, moderate intensity continuous training; MIIT, moderate intensity interval training; NC, non-compensators; NR, not reported; RT, resistance training; ↑, increase; ↓, decrease; ↔, no change.

Table S4. Moderator and subgroup analyses pre-post changes in daily energy intake in exercise groups only

Moderator	Moderator levels	p-value for between subgroup heterogeneity	Mean difference in kcal (95% CI)	p-value for within subgroup effect
Sex	Females (N=21)	0.381	-32 (-109, 45)	0.416
	Males (N=9)		-25 (-105, 55)	0.540
	Mixed (N=22)		-93 (-166, -21)	0.012
Dose/intensity	Low/Moderate (N=7)	0.656	44 (-35, 124)	0.273
	Moderate/High (N=7)		4 (-152, 161)	0.955
Energy intake method	Self-report (N=31)	<0.001[1]	-111 (-175, -47)	0.001
	Measured (N=7)		33 (-36, 103)	0.350
	Mixed (N=12)		-21 (-86, 43)	0.512
	DLW (N=2)		106 (67, 146)	<0.001

[1]If DLW effect sizes are removed, between subgroup heterogeneity p=0.010.
DLW, doubly-labelled water.

Table S5. Moderator and subgroup analyses for pre-post changes in daily and test meal energy intake combined in exercise groups only

Moderator	Moderator levels	p-value for between subgroup heterogeneity	Standardized mean difference (95% CI)	p-value for within subgroup effect
Sex	Females (N=21)	0.271	-0.060 (-0.214, 0.095)	0.450
	Males (N=13)		-0.018 (-0.140, 0.105)	0.777
	Mixed (N=25)		-0.148 (-0.255, -0.042)	0.006
Dose/intensity	Low/moderate (N=7)	0.821	0.014 (-0.161, 0.190)	0.158
	Moderate/High (N=7)		0.048 (-0.184, 0.279)	0.404
Energy intake type	Daily (N=52)	0.940	-0.096 (-0.180, -0.012)	0.025
	Single test meal (N=6)		-0.088 (-0.284, 0.108)	0.378
Energy intake method	Self-report (N=31)	<0.001[1]	-0.194 (-0.298, -0.090)	<0.001
	Measured (N=13)		0.019 (-0.080, 0.119)	0.700
	Mixed (N=12)		-0.044 (-0.179, 0.091)	0.520
	DLW (N=2)		0.446 (0.200, 0.692)	<0.001

[1]If DLW effect sizes are removed, between subgroup heterogeneity p=0.013.

DLW, doubly-labelled water.

k.beaulieu@leeds.ac.uk

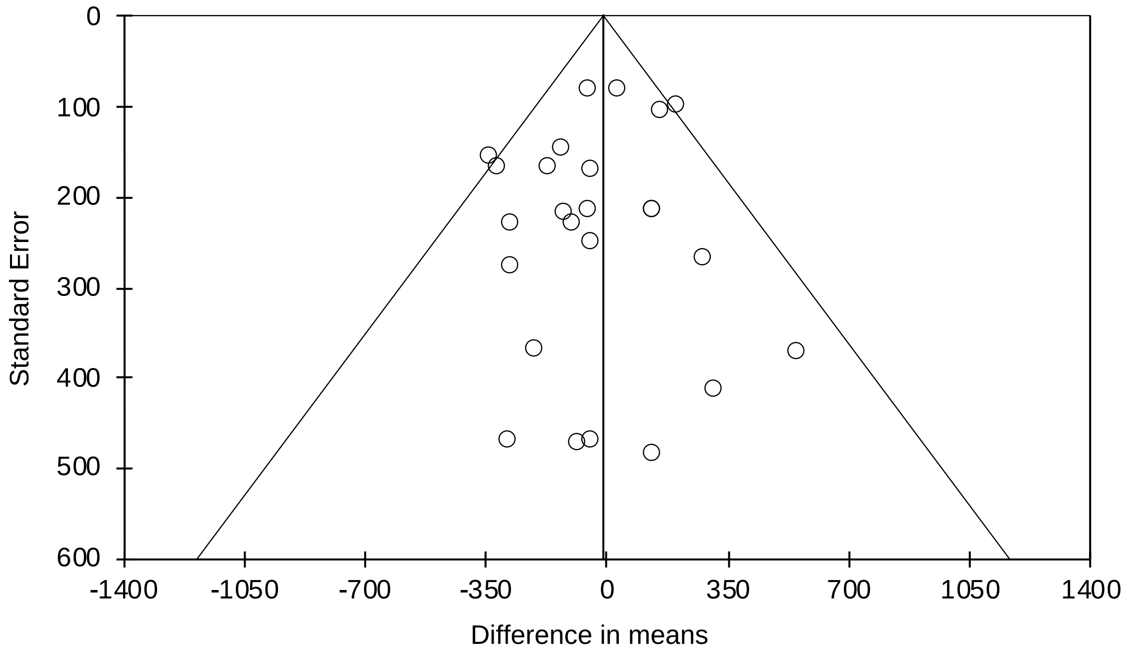

Figure S1. Funnel plot of post-intervention comparisons in daily energy intake between exerciser and no-exercise control groups (N=25 study arms).

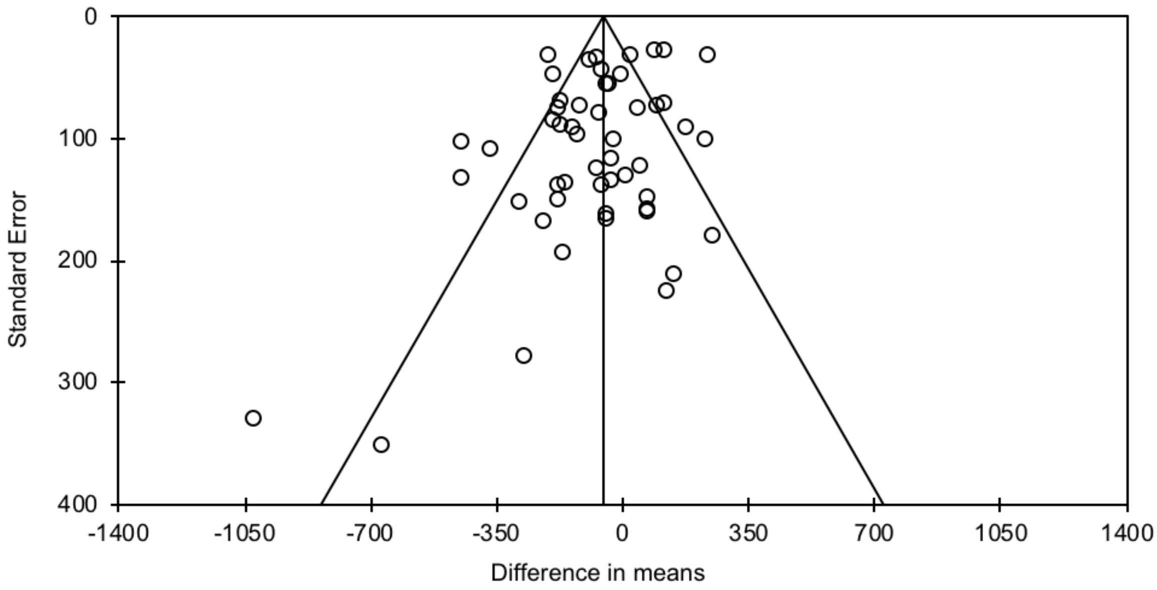

Figure S2. Funnel plot of pre-post changes in daily energy intake (N=52 study arms).

k.beaulieu@leeds.ac.uk

Group by Quality	Study name	Subgroup within study	Statistics for each study: Std diff in means	Lower limit	Upper limit	p-Value	Total
FairGood	Foster-Schubert et al. (2012)	Exercise alone	-0.324	-0.492	-0.155	0.000	114
FairGood	Martin et al. (2019)	20 KKW - adjusted DLW	0.526	0.159	0.893	0.005	26
FairGood	Martin et al. (2019)	20 KKW - buffet	-0.162	-0.515	0.191	0.369	25
FairGood	Martin et al. (2019)	8 KKW - adjusted DLW	0.380	0.049	0.711	0.025	30
FairGood	Martin et al. (2019)	8 KKW - buffet	-0.225	-0.555	0.104	0.180	29
FairGood	Riou et al. (2019)	Low (Day 1&2)	0.382	-0.165	0.930	0.171	11
FairGood	Riou et al. (2019)	Moderate (Day 1&2)	0.162	-0.396	0.720	0.570	10
FairGood	Rosenkilde et al. (2012)	High-dose - diet delivery pooled	-0.041	-0.626	0.543	0.890	9
FairGood	Rosenkilde et al. (2012)	Moderate-dose - diet delivery pooled	-0.049	-0.669	0.571	0.876	8
FairGood	Rosenkilde et al. (2013)	High-dose - test meal	0.141	-0.446	0.728	0.638	9
FairGood	Rosenkilde et al. (2013)	Moderate-dose - test meal	0.145	-0.442	0.733	0.628	9
FairGood	Manthou et al. (2010)	All	0.350	0.040	0.659	0.027	34
FairGood	Myers et al. (2019)		0.304	-0.008	0.616	0.056	24
**FairGood**			**0.114**	**-0.082**	**0.310**	**0.253**	**338**
Poor	Alizadeh et al. (2017)	Evening exercise	-0.053	-0.492	0.386	0.813	16
Poor	Alizadeh et al. (2017)	Morning exercise	-0.646	-1.066	-0.225	0.003	21
Poor	Bales et al. (2012)	Aerobic exercise	-0.164	-0.417	0.089	0.203	39
Poor	Bales et al. (2012)	Aerobic+Resistance exercise	-0.307	-0.561	-0.053	0.018	40
Poor	Bales et al. (2012)	Resistance exercise	-0.246	-0.504	0.013	0.062	38
Poor	Bhutani et al. (2013)	Exercise alone	-0.102	-0.461	0.257	0.578	24
Poor	Brandon & Elliot-Loyd (2006)	African American	1.755	1.034	2.477	0.000	15
Poor	Brandon & Elliot-Loyd (2006)	White	-0.598	-1.126	-0.070	0.026	13
Poor	Di Blasio et al. (2010)	Evening exercise	0.316	-0.148	0.780	0.181	15
Poor	Di Blasio et al. (2010)	Morning exercise	-0.171	-0.643	0.301	0.478	14
Poor	Donnelly et al. (2003)	Men	0.101	-0.338	0.541	0.652	16
Poor	Donnelly et al. (2003)	Women	-0.264	-0.621	0.092	0.146	25
Poor	Flack et al. (2018)	1500 kcal/wk	-0.247	-0.687	0.193	0.271	18
Poor	Flack et al. (2018)	3000 kcal/wk	-0.414	-0.668	-0.159	0.001	18
Poor	Halliday et al. (2017)	Pooled analysis	-0.132	-0.267	0.003	0.055	170
Poor	Heiston et al. (2019)	Continuous	-0.298	-0.776	0.181	0.223	14
Poor	Heiston et al. (2019)	Intermittent	-0.443	-0.934	0.048	0.077	14
Poor	Holliday et al. (2018)	Points-based physical activity	-0.908	-1.399	-0.417	0.000	18
Poor	Holliday et al. (2018)	Structured exercise	-0.084	-0.523	0.355	0.708	16
Poor	Jakicic et al. (2011)	Groups combined	-0.348	-0.462	-0.233	0.000	248
Poor	Kirkwood et al. (2007)	Exercise only	-0.477	-0.901	-0.053	0.028	19
Poor	Macias-Cervantes et al. (2015)	Exercise alone	-0.062	-0.531	0.407	0.797	14
Poor	Nieman et al. (1990)		-0.457	-0.892	-0.023	0.039	18
Poor	Quist et al. (2019)	Bike	-0.212	-0.656	0.231	0.348	16
Poor	Quist et al. (2019)	Moderate-intensity	0.014	-0.337	0.364	0.939	25
Poor	Quist et al. (2019)	Vigorous-intensity	0.091	-0.292	0.475	0.640	21
Poor	Reseland et al. (2001)	Exercise only	-0.160	-0.417	0.098	0.224	47
Poor	Rhew et al. (2007)		-0.006	-0.210	0.197	0.951	74
Poor	Washburn et al. (2012)		0.067	-0.244	0.377	0.673	32
Poor	Washburn et al. (2015)	Men - 400 kcal/session	-0.238	-0.800	0.324	0.407	10
Poor	Washburn et al. (2015)	Men - 600 kcal/session	0.195	-0.364	0.755	0.494	10
Poor	Washburn et al. (2015)	Women - 400 kcal/session	-0.068	-0.623	0.487	0.810	10
Poor	Washburn et al. (2015)	Women - 600 kcal/session	0.141	-0.416	0.698	0.620	10
Poor	Willis et al. (2019)	Early exercise	-0.222	-0.783	0.339	0.438	10
Poor	Willis et al. (2019)	Late exercise	0.477	-0.108	1.062	0.110	10
Poor	Willis et al. (2019)	Sporadic exercise	-0.448	-1.029	0.134	0.131	10
Poor	Caudwell et al. (2013b)	Men	0.077	-0.163	0.316	0.530	35
Poor	Caudwell et al. (2013b)	Women	-0.048	-0.215	0.119	0.572	72
Poor	Cornier et al. (2012)		-0.322	-0.841	0.197	0.224	12
Poor	Crampes et al. (2003)		-0.306	-0.847	0.235	0.268	11
Poor	Garnier et al. (2015)		0.073	-0.085	0.232	0.364	156
Poor	Alkahtani et al. (2014)	HIIT	-0.217	-1.010	0.576	0.592	5
Poor	Alkahtani et al. (2014)	MIIT	0.391	-0.422	1.205	0.346	5
Poor	Damour et al. (2019)	Exercise anytime (MealEx)	-0.845	-1.866	0.176	0.105	4
Poor	Damour et al. (2019)	Exercise before meal (ExMeal)	-1.377	-2.601	-0.154	0.027	4
**Poor**			**-0.150**	**-0.233**	**-0.067**	**0.000**	**1432**
**Overall**			**-0.109**	**-0.186**	**-0.033**	**0.005**	**1770**

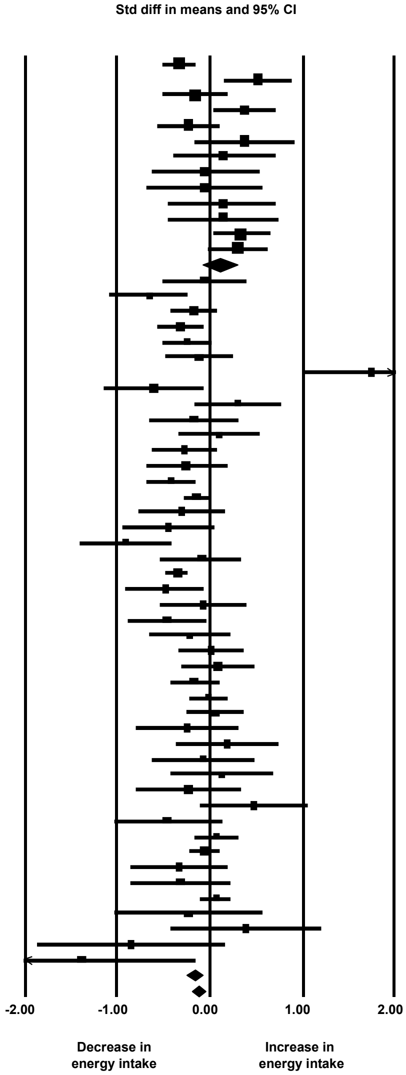

Figure S3. Forest plot of pre-post changes in daily and test meal energy intake in individuals with overweight or obesity, grouped by study quality (N=58 study arms).

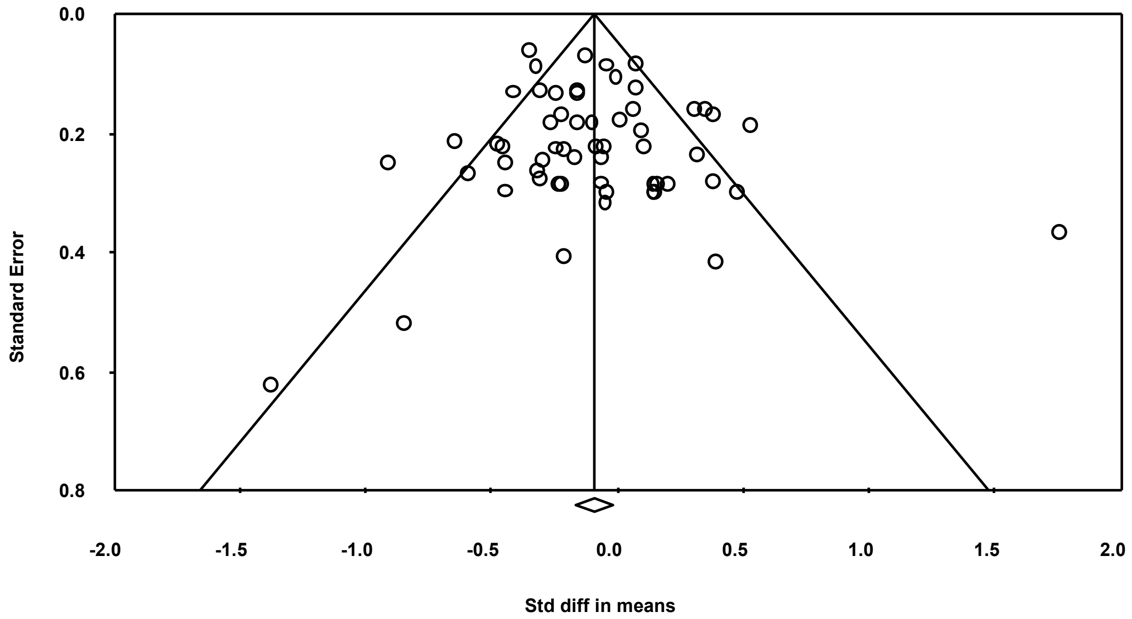

Figure S4. Funnel plot of pre-post changes in daily and test meal energy intake (N=58 study arms).

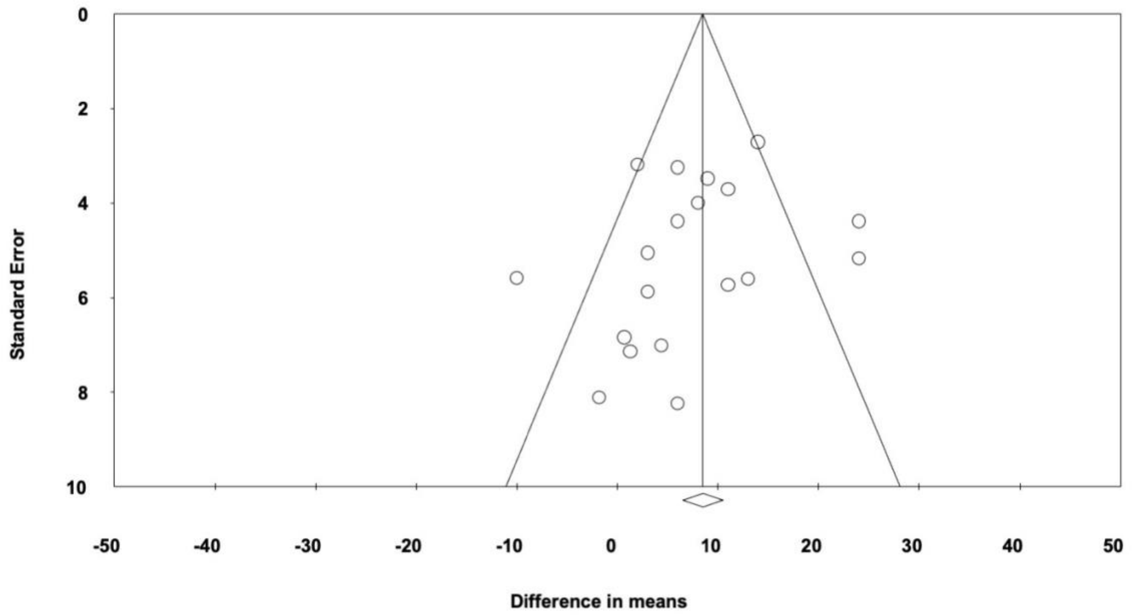

Figure S5. Funnel plot of pre-post changes in fasting hunger (N=19 study arms)

Study name	Subgroup within study	Statistics for each study				
		Difference in means	Lower limit	Upper limit	p-Value	Total
Guelfi et al. (2013)	Aerobic	15	7	23	0.000	12
Guelfi et al. (2013)	Resistance	11	0	22	0.050	13
Heiston et al. (2019)	Continuous	4	-7	14	0.467	14
Heiston et al. (2019)	Intermittent	-0	-8	8	0.980	14
Martins et al. (2017)	1/2 HIIT	1	-9	11	0.845	9
Martins et al. (2017)	HIIT	-7	-16	2	0.115	13
Martins et al. (2017)	MICT	-3	-15	9	0.615	13
Quist et al. (2019)	Bike	-1	-7	5	0.758	19
Quist et al. (2019)	Moderate-intensity	4	-2	10	0.197	30
Quist et al. (2019)	Vigorous-intensity	-3	-11	5	0.434	24
Riou et al. (2019)	Low-intensity	3	-10	15	0.689	11
Riou et al. (2019)	Moderate-intensity	-4	-21	12	0.621	8
Rosenkilde et al. (2013)	High-dose	16	10	22	0.000	18
Rosenkilde et al. (2013)	Moderate-dose	-5	-10	0	0.071	18
Kanaley et al. (2014)		6	1	11	0.028	13
Martins et al. (2010)		-16	-22	-10	0.000	15
Myers et al. (2019)		-3	-10	4	0.367	24
		**1**	**-3**	**5**	**0.641**	**268**

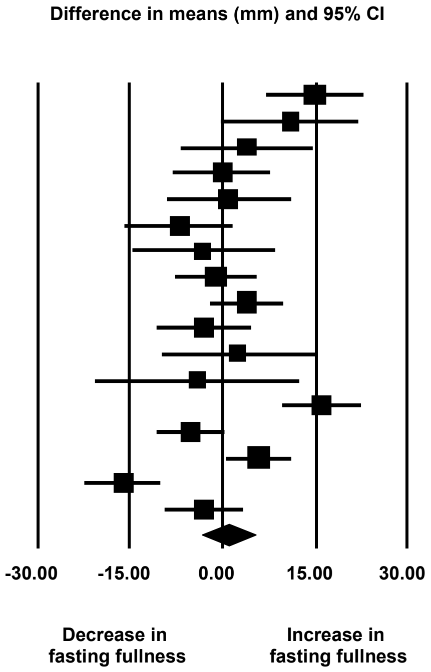

Figure S6. Forest plot of changes in fasting fullness showing no overall change after exercise training in individuals with overweight or obesity (N=17 study arms).

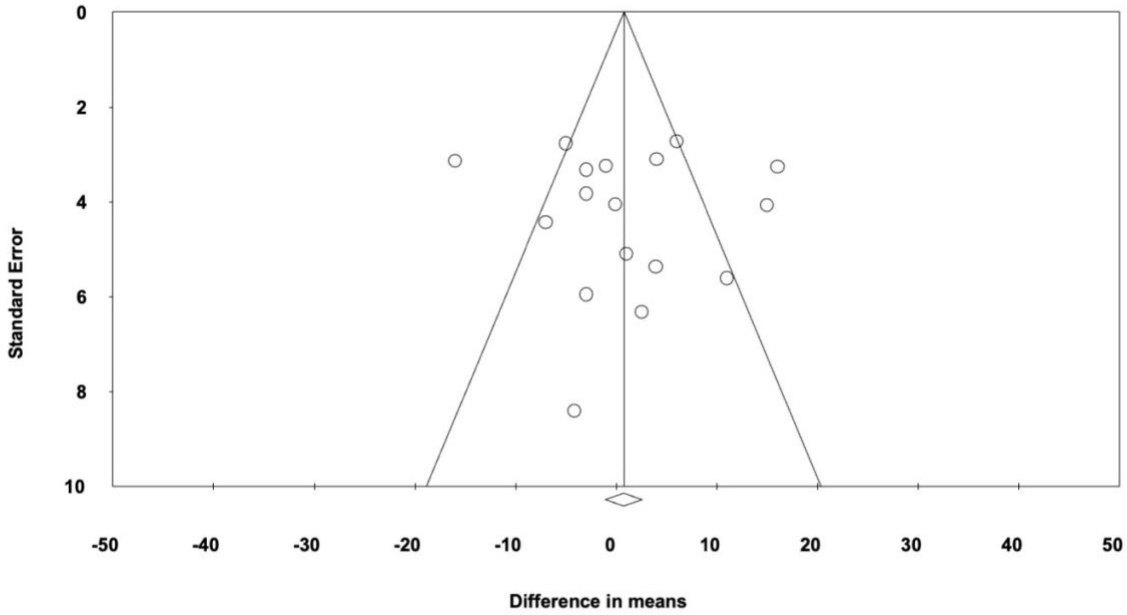

Figure S7. Funnel plot of pre-post changes in fasting fullness (N=17 study arms)

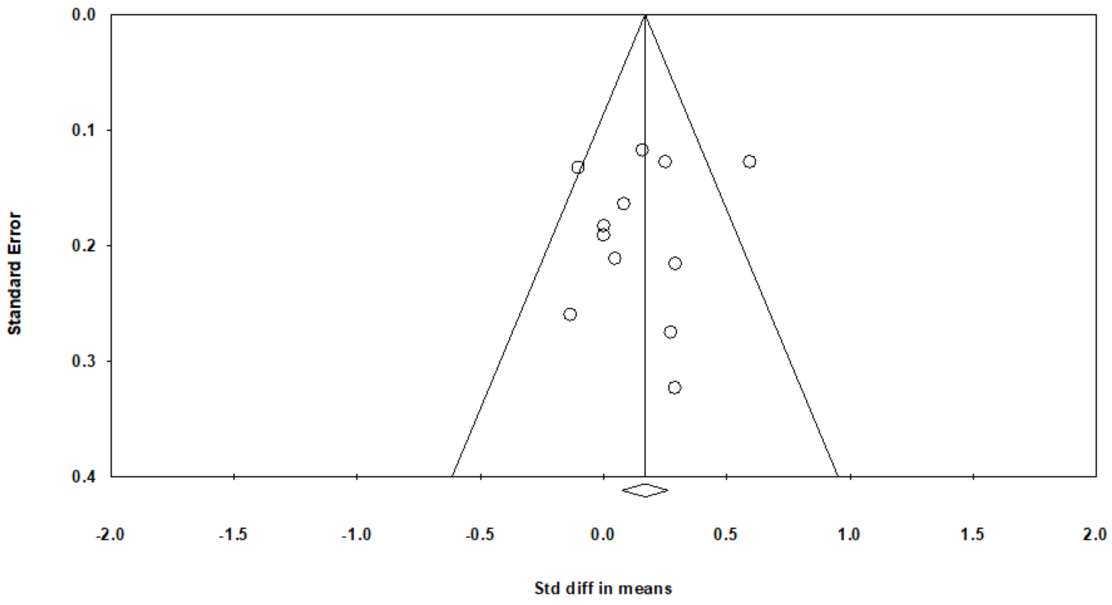

Figure S8. Funnel plot of pre-post changes in dietary restraint (N=12 study arms)

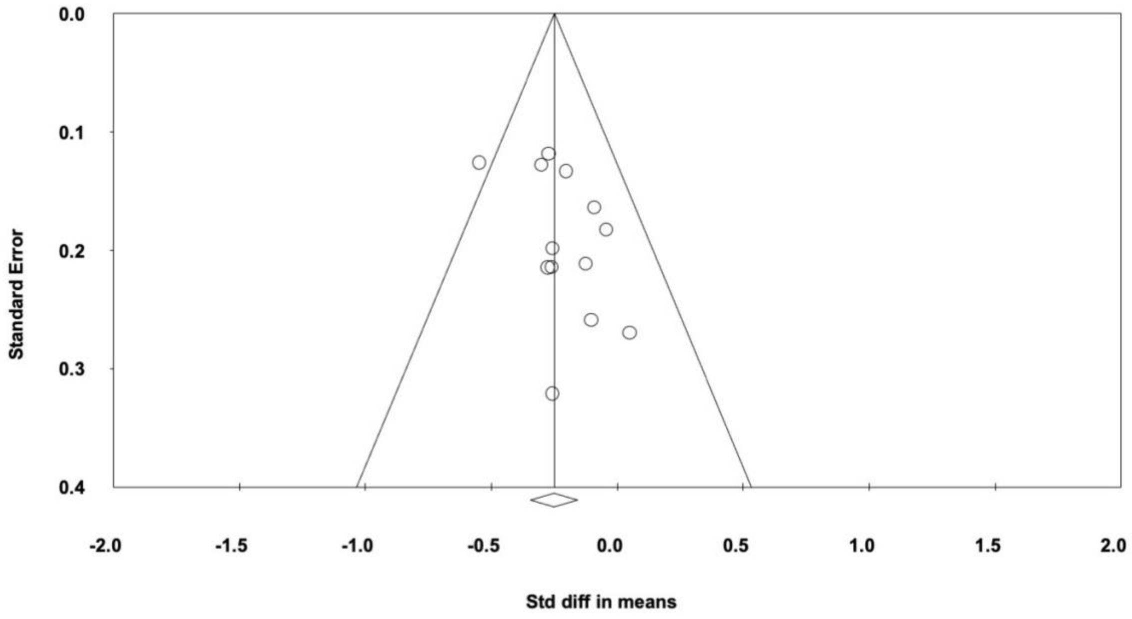

Figure S9. Funnel plot of pre-post changes in disinhibition/uncontrolled eating (N=13 study arms)

k.beaulieu@leeds.ac.uk

Study name	Subgroup within study	Statistics for each study: Std diff in means	Lower limit	Upper limit	p-Value	Total
Beaulieu et al. (2020)		-0.056	-0.315	0.202	0.670	46
Martin et al. (2019)	20KKW	-0.066	-0.312	0.179	0.597	51
Martin et al. (2019)	8KKW	0.065	-0.164	0.293	0.577	59
Quist et al. (2019)	Bike	-0.328	-0.752	0.096	0.130	18
Quist et al. (2019)	Moderate-intensity	0.203	-0.121	0.526	0.219	30
Quist et al. (2019)	Vigorous-intensity	-0.240	-0.619	0.139	0.215	22
Riou et al. (2019)	Low-intensity	0.769	0.167	1.370	0.012	11
Riou et al. (2019)	Moderate-intensity	0.429	-0.219	1.077	0.194	8
Rosenkilde et al. (2013)	High-dose	-0.036	-0.449	0.377	0.864	18
Rosenkilde et al. (2013)	Moderate-dose	-0.290	-0.712	0.132	0.178	18
Bryant et al. (2012)		-0.056	-0.287	0.174	0.632	58
		**-0.014**	**-0.142**	**0.114**	**0.831**	**339**

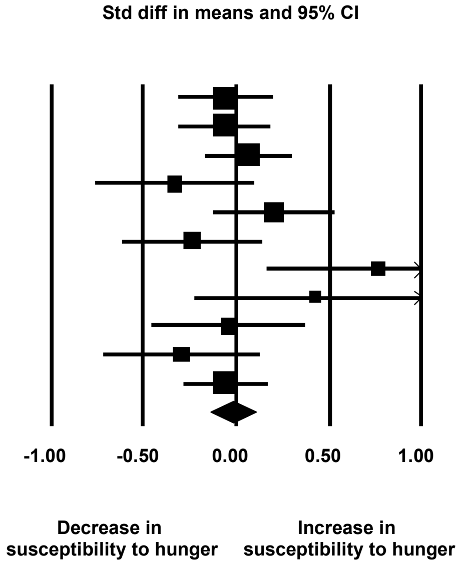

Figure S10. Forest plot of changes in susceptibility to hunger showing no overall change after exercise training in individuals with overweight or obesity (N=11 study arms).

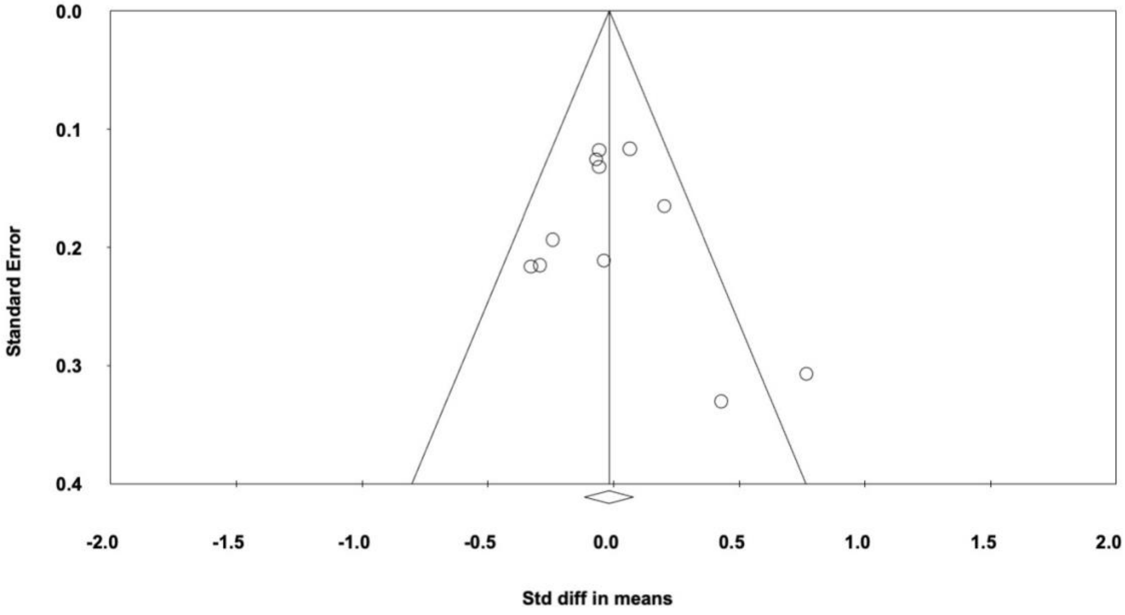

Figure S11. Funnel plot of pre-post changes in susceptibility to hunger (N=11 study arms)
